# Signaling pathways related to interstitial cystitis

**DOI:** 10.3389/fimmu.2026.1774072

**Published:** 2026-04-23

**Authors:** Haowen Wang, Shuang Liu, Yuan Sun, Lina Wang

**Affiliations:** 1 Department of Urology, First Affiliated Hospital of Dalian Medical University, Dalian, China; 2 Liaoning Laboratory of Cancer Genomics and Department of Cell Biology, Dalian Medical University, Dalian, China

**Keywords:** bladder pain syndrome, fibrosis, IC/BPS, inflammation, interstitial cystitis, mast cell, neurogenic inflammation, signaling pathway

## Abstract

Interstitial cystitis/bladder pain syndrome (IC/BPS) represents a chronic, non-infectious inflammatory bladder disorder, predominantly affecting women. Primary symptoms include ongoing pelvic pain, frequent urination, and urgency, often accompanied by functional somatic pain syndromes and psychological health disturbances. The severity of IC symptoms significantly impairs patients’ quality of life, highlighting the necessity for research on the pathogenesis, progression, and treatment of IC/BPS. Although the exact etiology of IC remains unclear, and there is no simple or definitive diagnostic method, current clinical treatments primarily focus on symptom relief rather than a cure. In recent years, extensive experimental studies on the etiology and pathophysiology of IC have identified a multitude of signaling pathways involved in the disease’s initiation, progression, and self-repair mechanisms. This study focuses on IC-related signaling pathways, analyzing the role of key molecular signals in the onset and progression of IC. By integrating clinical symptoms and signs with molecular biology insights, we provide a comprehensive overview and comparative analysis of IC’s etiology and pathophysiological activities. The goal is to establish a theoretical foundation for the development of more efficient diagnostic tools, drug therapies, treatment evaluations, and preventive strategies for IC.

## 1 Introduction

Interstitial cystitis (IC), or bladder pain syndrome (BPS), is a chronic non-infectious inflammatory bladder condition. Key symptoms include urgency, frequency, nocturia, and bladder/pelvic pain or pressure. These often coexist with somatic pain disorders (e.g., fibromyalgia) and psychological conditions like depression. Pathological hallmarks are bladder wall fibrosis, inflammatory cell infiltration (lymphocytes, neutrophils), and distinctive mast cell infiltration (2017). In 1914, Hunner described the classic Hunner’s ulcer—intersecting fibrotic tissue appearing as reddish areas with fibrin or scarring. Based on this, IC is subclassified into Hunner type (HIC) and non-Hunner type (NHIC) (1).

This classification holds significant clinical and pathophysiological implications. HIC is typically characterized by discrete ulcers or scars (Hunner’s lesions) visible under cystoscopy, accompanied by more pronounced inflammatory cell infiltration, epithelial denudation, and neovascularization. In contrast, NHIC lacks typical ulcerative lesions and mainly presents with diffuse petechial hemorrhage (glomerulations) after bladder hydrodistention. Its symptoms may be more focused on widespread pelvic pain and are more closely associated with central sensitization. A growing consensus suggests that HIC and NHIC may represent two distinct disease entities or different stages within a disease spectrum, with partially distinct etiological mechanisms and treatment responses (2, 3). Therefore, when investigating the signaling pathways of IC/BPS, it is crucial to differentiate the clinical phenotype from which the evidence originates.

Historically, diagnosis relied on cystoscopic identification of Hunner’s ulcers or mucosal tears/bleeding after hydrodistension (80–100 cmH_2_O for 1–2 minutes). This strict criterion led to underdiagnosis of many NHIC patients presenting primarily with pelvic pain. In 2008, the European Society for the Study of Interstitial Cystitis (ESSIC) defined BPS as “pelvic discomfort related to the bladder, plus urinary symptoms, lasting >6 months after excluding other causes” (van de Merwe et al., 2008). Later guidelines increasingly use “BPS” as a primary pain syndrome linked to bladder symptoms. However, experts note HIC may be a distinct entity with specific endoscopic findings and treatment responses. Some equate BPS more with NHIC. While ESSIC prefers “BPS,” they acknowledge omitting “IC” could impede research; hence “BPS/IC” is often used. The AUA uses “IC/BPS” synonymously. International bodies recommend “BPS” for symptom-based diagnosis but suggest reserving “IC” for patients with Hunner’s lesions due to its distinct pathology (2, 4). The AUA’s 2022 definition requires symptoms (urgency, pain) lasting ≥6 weeks with negative urine culture (5).

Prevalence ranges from 0.01% to 2.3%, affecting women ~5 times more than men (2). U.S. surveys found 2.7%-6.5% of women report IC/BPS-like symptoms (6). Using high-sensitivity criteria, prevalence was 4.2%; high-specificity criteria (including pain on filling and no antibiotic relief) yielded 1.9% (7). IC significantly reduces quality of life and is associated with depression and anxiety.

Etiology remains unclear, with leading hypotheses including: 1) autoimmune mechanisms, focusing on mast cell activation (8, 9); 2) neurogenic inflammation, involving receptor and central sensitization (10); and 3) urothelial injury, featuring glycosaminoglycan (GAG) layer damage and increased permeability (11 1994). Diagnosis is challenging due to ambiguous definitions, unclear etiology, and lack of simple accurate methods, leading to misdiagnosis and underdiagnosis (12–14).

This review analyzes signaling pathways in IC/BPS related to epithelial injury/repair, autoimmune responses, and neurogenic inflammation. It explores their roles in disease pathogenesis to elucidate its complex, multifactorial nature. Integrating molecular biology with clinical manifestations, it aims to outline etiology and pathophysiology, providing a theoretical basis for improved diagnostics, targeted therapies, and treatment assessment.

## 2 Literature search and analysis methods

To systematically review the key signaling pathways related to interstitial cystitis/bladder pain syndrome (IC/BPS), this study, adhering to the principles of a narrative review, employed the following strategies for literature retrieval, screening, and synthesis.

We systematically searched two major literature databases, PubMed and Web of Science, for relevant publications and their cited references from the past 20 years. The search keyword combinations were constructed based on disease names and core pathological mechanisms, primarily including: “interstitial cystitis”, “bladder pain syndrome”, “IC/BPS”, “signaling pathway”, “inflammation”, “urothelium”, “mast cell”, “fibrosis”, “neurogenic inflammation”, and their related terms.

We primarily included original research (encompassing both basic and clinical studies) that investigated specific signaling pathways in the pathophysiology of IC/BPS. Inclusion criteria were: (a) Study subjects included tissue samples from IC/BPS patients, IC/BPS animal models, or relevant *in vitro* cell models; (b) The study explicitly explored the mechanism of action of a signaling pathway in IC/BPS; (c) Article type was original research or high-quality review (used for background information); (d) Publication language was English. Exclusion criteria included: case reports, conference abstracts, and purely clinical descriptive studies not focused on specific signaling pathway mechanisms.

In the process of evidence integration, we assigned different weights to studies at different levels. Findings from studies using bladder tissue from IC/BPS patients were considered the most direct evidence and given the highest weight. Animal model studies provided evidence for causality in mechanisms, used to elucidate the role of pathways within an intact physiological system. *In vitro* cell studies were utilized to reveal detailed molecular-level interactions. When evidence from different types of studies pointed in the same direction, the strength of the conclusion was considered higher.

When encountering conflicting research results, we attempted to analyze and elucidate them from the following aspects: (a) Differences in study design: Such as patient subtypes (Hunner type vs. non-Hunner type), types of animal models (e.g., cyclophosphamide-induced, autoimmune models), or differences in cell lines; (b) Methodological details: Such as differences in the sensitivity and specificity of detection methods or intervention approaches; (c) Biological context: Such as different stages of the disease or the dual roles of pathways in different cell types. These inconsistencies are explicitly pointed out in the text, and the most plausible explanations are discussed within the current overall framework of understanding IC/BPS pathophysiology.

## 3 Signaling pathways related to epithelial injury and repair

### 3.1 Epithelial injury caused by ischemia and hypoxia

In patients with IC/BPS, the bladder is subjected to an ischemic and hypoxic environment. Michel A. et al. observed that bladder blood flow perfusion in IC/BPS patients decreases with bladder filling, whereas perfusion in normal bladders increases with filling (15). A decline in bladder blood flow perfusion among interstitial cystitis patients causes an oxygen imbalance in the bladder, resulting in tissue hypoxia and ischemic-hypoxic damage (16, 17). This also provides a theoretical foundation for hyperbaric oxygen therapy in the treatment of IC/BPS.

In IC, the bladder experiences intermittent hypoxia during the repetitive transitions between filled and empty states. The recurring hypoxia triggers the production of reactive oxygen species (ROS) in the cells of bladder tissue (18). ROS are well-established contributors to oxidative stress, capable of causing direct damage to cellular structures and DNA. These species are generated from electron transfer within the oxidative phosphorylation chain and through the catalytic action of NADPH oxidase (NOX), exhibiting strong oxidizing properties. Recent studies have identified ROS as key regulators of various cellular signaling pathways, functioning through redox-based mechanisms (19). Notably, Oliveira et al. demonstrated that ROS levels were significantly elevated in bladder cells of mice subjected to CYP-induced IC models. Furthermore, the application of antioxidants to scavenge ROS resulted in a marked alleviation of symptoms, including urinary frequency and pain sensitivity, in the affected animal models (20). The ischemia-hypoxia response in IC is predominantly regulated by the hypoxia-inducible factor-1 (HIF-1) signaling pathway and its downstream VEGF pathway, causing bleeding and ulcers in the bladder mucosa. Notably, the HIF-1 signaling pathway and its downstream products can counteract oxidative molecules in the bladder, thereby preventing further oxidative stress damage to the tissue. Consequently, a complete blockade of the HIF-1α pathway is not a feasible strategy; instead, the timely alleviation of ischemia-hypoxia in the bladder or the clearance of excessive reactive oxygen species (ROS) accumulation represents the primary therapeutic approach for IC.

#### 3.1.1 HIF-1 signaling pathway

**Figure 1:**
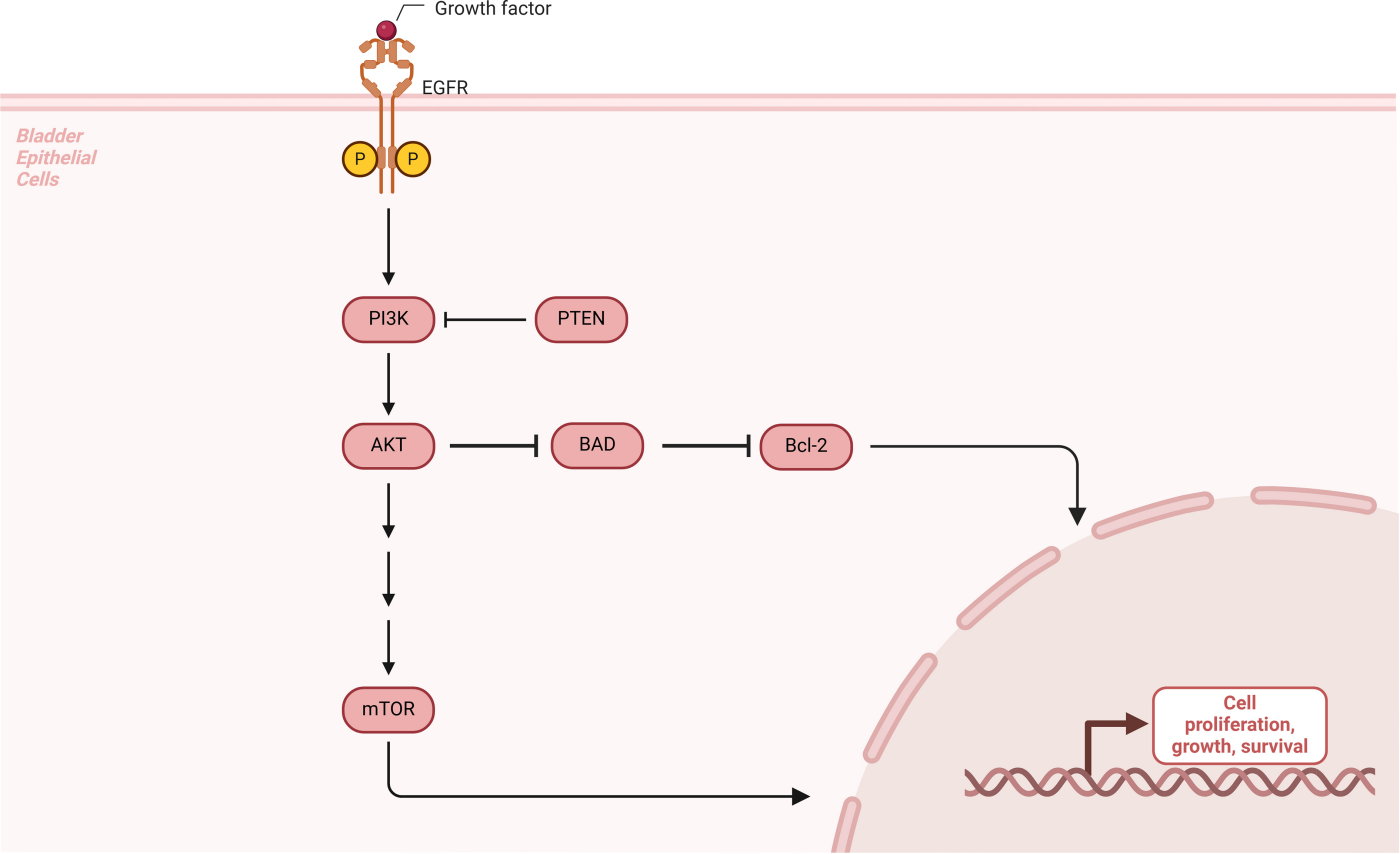
The diagram illustrates the PI3K-Akt/mTOR signaling pathway and its role in promoting cell proliferation, survival, and repair in interstitial cystitis (IC). Growth factors such as EGF bind to their receptors (EGFR), activating PI3K, which phosphorylates Akt. Activated Akt inhibits pro-apoptotic proteins like BAD and promotes anti-apoptotic proteins like Bcl-2, enhancing cell survival. Akt also activates mTOR, which stimulates cell proliferation, growth, and tissue repair through downstream transcription factors like HIF-1α. In IC, this pathway plays a key role in repairing damaged bladder urothelium, reducing apoptosis, and mitigating inflammation, thus contributing to bladder epithelial regeneration and fibrosis prevention.

As a transcription factor, hypoxia-inducible factor-1 (HIF-1) plays a vital role in oxygen regulation and is formed by two subunits: HIF-1α and HIF-1β. During normoxia, HIF-1α is hydroxylated at particular proline residues, leading to its ubiquitination and degradation, which inhibits its transcriptional activity. Conversely, under hypoxic conditions, HIF-1α stabilizes, pairs with HIF-1β, and moves to the nucleus, where it activates transcription, increasing the expression of downstream molecules like VEGF, fostering new capillary growth, lowering oxygen consumption, improving oxygen delivery, and mitigating tissue hypoxia. (21–24). Additionally, it can lower ROS levels and inhibit the generation of hypoxia-induced apoptotic factors, thereby reducing hypoxic apoptosis and pyroptosis in epithelial cells (**Figure 1**).

HIF-1α is highly expressed in the bladder of IC patients. Lee et al. found that HIF-1α protein expression in bladder biopsies from IC patients was 2. 3 times higher than that of the control group (patients with stress urinary incontinence without bladder mucosal damage) (17). Through paired lesion (areas with red hemorrhagic spots)/non-lesion biopsies in 25 patients with Hunner-type interstitial cystitis, RNA sequencing revealed extensive transcriptomic changes in the lesion areas, with significant upregulation of 24 pro-survival/adaptation pathways such as the HIF1α signaling pathway, PI3K-Akt signaling pathway, RAS signaling pathway, and MAPK signaling pathway. The mRNA and protein levels of HIF1α, as well as the mRNA levels of IFN-γ and IL-2, were specifically elevated in the lesion areas, while most other inflammatory factor levels and typical histopathological features (such as the degree of inflammatory cell infiltration, epithelial denudation, and T-cell subset proportions) showed no significant differences between lesion and non-lesion areas. This provides deeper insights into the strong correlation between IC/PBS with Hunner lesions and the HIF-1α signaling pathway (3). Prior to this, Lee et al. included 32 IC patients and 8 healthy volunteers as controls. Bladder tissue samples were collected from both groups. Western blot and immunostaining analyses showed that the expression levels of both HIF-1α and VEGF proteins were significantly higher in the study group compared to the control group. The relative intensities of HIF-1α were (60.60 ± 7.81) vs. (26.20 ± 4.72), and those of VEGF were (43.60 ± 5.37) vs. (20.25 ± 1.45), respectively. Confocal microscopy revealed that overexpression of VEGF in the study group’s biopsy samples was particularly prominent in umbrella cells. These molecular events may be associated with the formation mechanism of glomerulations during hydrodistention in IC bladders (17). In IC/PBS, Hunner lesions are characterized by hemorrhagic spots. Therefore, the studies mentioned above collectively indicate that the upregulation of the HIF-1 signaling pathway is more strongly correlated with the Hunner ulcer phenotype.

The activation of the HIF-1 signaling pathway reduces intracellular oxidative levels and mitigates ROS-induced hypoxic apoptosis and pyroptosis (25). Guo et al. constructed a hypoxic cell model by knocking down HIF-1α with siRNA and subjecting the cells to 95% N_2_ and 5% CO_2_ for 15 minutes. They measured the ratio of glutathione to oxidized glutathione (GSH/GSSG) to assess intracellular redox levels. The GSH/GSSG ratios in the HIF-1α knockdown group and the non-knockdown group were 100% and 59%, respectively, demonstrating that HIF-1 gene knockdown exacerbated the oxidative environment within the cells, leading to increased oxidative apoptosis. The expression of two essential pentose phosphate pathway enzymes, glucose-6-phosphate dehydrogenase (G6PD) and phosphogluconate dehydrogenase (PGD), significantly declined in the HIF-1α knockdown group. The pentose phosphate pathway directly oxidizes and dehydrogenates glucose, producing substantial NADPH for cellular reduction reactions. A significant decline in intracellular NADPH levels was observed in the HIF-1α knockdown group compared to the control, highlighting HIF-1’s role in boosting key pentose phosphate pathway enzymes to maintain reducing substances and mitigate oxidative stress within cells. In the hypoxic cell group without HIF-1α knockdown, inhibiting HIF-1α protein hydrolysis using hydroxylase inhibitors significantly reduced oxidative levels, with GSH/GSSG rising to 118. 2%. Moreover, treatment with the antioxidant N-acetyl-L-cysteine (NAC) and hydrogen peroxide increased cell survival under hypoxic conditions, reducing cell death rates from 64. 3 ± 5. 2% to 32. 1 ± 5. 0%, highlighting the crucial role of the HIF-1 pathway in maintaining redox homeostasis (26). In the experiments *in vitro* with uroepithelial cells (SV40 T-antigen immortalized human uroepithelial cells-1, SV-HUC-1), Wu et al. exposed cells to a hypoxic environment for 3 hours and then returned them to normoxic conditions. They found that HIF-1α expression significantly increased during ischemia and hypoxia, and flow cytometry analysis revealed a marked increase in both early and late apoptotic cells (p < 0. 001). The addition of the antioxidant NAC, which scavenged and inhibited ROS production, significantly reduced the number of late apoptotic cells, while the expression of hypoxia and apoptosis-related factors such as HIF-1α, BAX, and caspase-3 decreased markedly. This evidence supports the involvement of the HIF-1 pathway in ROS-mediated hypoxic apoptosis induced by tissue hypoxia (27).

The aforementioned studies indicate that HIF-1α expression is generally upregulated in the bladders of IC/PBS patients, but it is particularly pronounced in the HIC subtype. Its expression intensity is directly correlated with the severity of tissue damage in ulcerated areas, such as petechial hemorrhage and mucosal denudation (3) (17). This suggests that the sustained activation of the HIF-1 pathway may be closely related to the characteristic mucosal defects, chronic ischemia-hypoxia, and neovascularization observed in HIC. In contrast, the activation pattern of this pathway and its association with symptoms (such as diffuse pain) in non-Hunner type IC (NHIC) require further subtype-specific investigation. Currently, evidence regarding the role of HIF-1 in antioxidant and anti-apoptotic processes primarily derives from *in vitro* hypoxic cell models, and its precise role in human IC subtypes remains to be validated (26).

#### 3.1.2 VEGF signaling pathway

**Figure 2:**
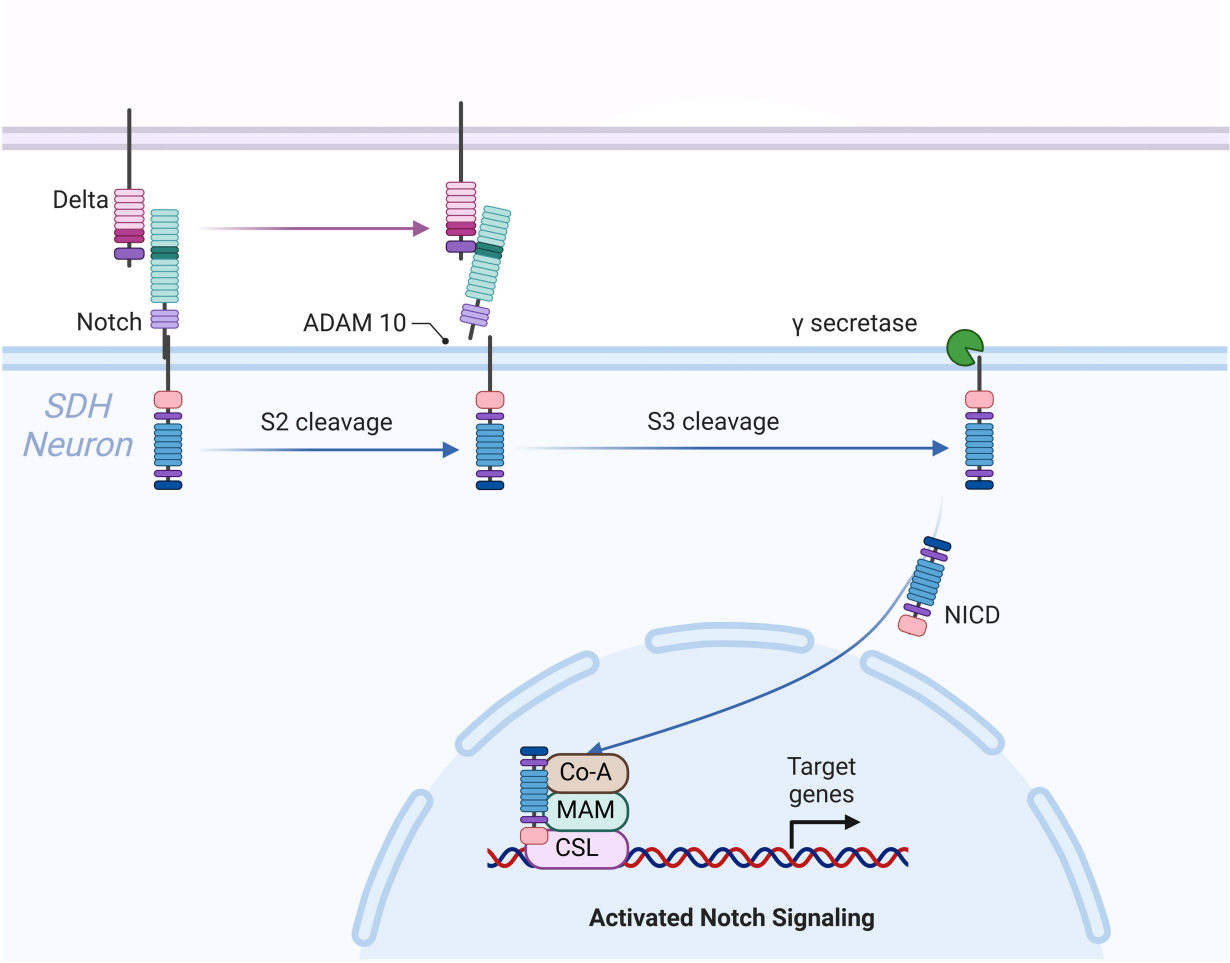
The diagram illustrates the Notch signaling pathway and its role in neuroinflammation and mechanical allodynia associated with interstitial cystitis/bladder pain syndrome (IC/BPS). Notch signaling is initiated when the Delta ligand binds to the Notch receptor, triggering S2 cleavage by ADAM10 and S3 cleavage by γ-secretase. This releases the Notch intracellular domain (NICD), which translocates to the nucleus to activate target genes. In IC, Notch1 upregulation in the spinal dorsal horn (SDH) promotes microglial activation and inflammatory cytokine production (e.g., TNF-α, IL-1β), reducing pain thresholds and contributing to pelvic pain. Inhibition of Notch1 with γ-secretase inhibitors alleviates neuroinflammation and pain.

As a downstream pathway of HIF-1, the vascular endothelial growth factor (VEGF) pathway is governed by HIF-1 as a key transcription regulator. Activation of HIF-1 under hypoxic conditions leads to increased expression of VEGF. During tissue hypoxia, the activation of HIF-1 results in the overexpression of VEGF, facilitating the formation of new blood vessels to enhance blood and oxygen supply to the tissues. However, these newly formed capillaries are relatively fragile, and the increased vascular permeability can lead to exudation and rupture (28), this is likely one of the reasons for the petechial bleeding observed in the bladders of IC patients following hydrodistention (17). In IC, VEGF serves as a factor that regulates oxygen levels and promotes angiogenesis, thereby improving tissue perfusion and alleviating tissue hypoxia (15, 29). Excessive VEGF/VEGFR expression has been found to correlate with bladder pain and damage to the epithelium (**Figure 2**).

Saban et al. discovered that VEGF and VEGF receptors (VEGFR) are highly expressed in the bladder tissues of patients with IC/BPS, with VEGFR primarily expressed in the bladder urothelium and ganglia (30). Lee et al. conducted a clinical study with 32 IC patients (26 females and 6 males) and collected biopsy samples from petechial bleeding areas after bladder hydrodistention. Immunoblotting and immunohistochemical analyses showed a significant increase in VEGF protein expression in the experimental group compared to the control (43. 60 ± 5. 37 vs. 20. 25 ± 1. 45, P < 0. 05). CK7 staining confirmed VEGF overexpression in the umbrella cells of the urothelium’s outermost layer in the samples (17). In the bladder, both VEGF and its receptors are expressed in the urothelium, the lamina propria, and the ganglion cells of the bladder wall (29, 31). The high expression of VEGF can prevent ischemia-induced tissue damage to the bladder epithelium and corresponding neurons (32). Tamaki et al. evaluated the expression of VEGF receptors (VEGFR) in bladder biopsies from IC patients, finding significantly higher expression of VEGFR in the urothelial cells, subepithelial blood vessels, and wall ganglia of IC bladders compared to normal bladders (p < 0. 05) (33).

Lai et al. pretreated mice with the VEGF antibody B20–4 before intraperitoneally injecting cyclophosphamide (CYP) to establish an animal model. Behavioral experiments showed that anti-VEGF pretreatment reduced CYP-induced pelvic pain hypersensitivity, with pain thresholds in the von Frey filament test returning to levels similar to the control group (p < 0. 05). This indicates that systemic treatment with anti-VEGF neutralizing antibodies effectively alleviated pelvic/bladder pain in the cystitis model in mice (34). Similarly, Shin et al. found that treatment with the anti-angiogenic tyrosine kinase inhibitor axitinib effectively alleviated bladder damage induced by HCl. The treatment group showed a substantial suppression of VEGF/VEGFR expression in the bladder, which led to reduced bladder pain, improved epithelial integrity, and a notable decline in urinary frequency (p < 0. 05) (35). This research highlights new directions and targets for clinical IC therapy.

The elevated expression of VEGF/VEGFR in the bladder tissue of IC/BPS has been confirmed by multiple studies. Notably, the study samples from Lee et al. were taken from the petechial hemorrhage areas after hydrodistention, which is a common manifestation of IC (particularly in cases diagnosed via hydrodistension), but can occur in both HIC and NHIC. On the other hand, Tamaki et al. found that VEGF expression in the bladder urothelium, blood vessels, and ganglia of IC patients was higher than normal, though subtypes were not clearly distinguished. Intervention studies targeting VEGF (such as anti-VEGF antibody B20–4 and axitinib) have demonstrated effects in alleviating bladder pain and damage in animal models (34, 35). Overall, the VEGF pathway, as a downstream target of HIF-1, likely plays a central role in ulcer formation, abnormal angiogenesis, and associated pain in HIC. Its disruption of tight junctions (such as ZO-1), increasing epithelial permeability, may be one of the mechanisms underlying urine solute permeation, neural sensitization, and pain in NHIC. However, more research focusing on specific subtypes of patients is needed to clarify the relative importance of the VEGF pathway in HIC and NHIC.

### 3.2 Epithelial injury induced by disruption of cell junctions

Tight junctions play a critical role in forming a barrier between the bladder urothelium and the underlying epithelium, as well as between urine and its solutes. When the bladder loses its normal impermeability, a condition referred to as epithelial leakage occurs. This leakage allows potassium ions to diffuse into the bladder wall, leading to depolarization of sensory nerves and muscle tissues, which results in tissue damage. Consequently, this process contributes to symptoms such as pelvic pain, urgency, and frequency of urination (29).

#### 3.2.1 VEGF signaling pathway

VEGF, commonly known as Vascular Permeability Factor (VPF), has been shown to significantly contribute to the increase in vascular permeability. The overexpression of VEGF contributes to the injury of the bladder epithelium by enhancing vascular permeability. A range of adhesion molecules regulates the integrity of endothelial cell junctions and vascular barriers by forming tight, gap, and adherens junctions (36), VEGF acts through VEGFR2 and Src kinase to phosphorylate junctional proteins, disrupting gap junction communication between adjacent endothelial cells, leading to the breakdown of cell-cell junctions and subsequently increased epithelial permeability, which exacerbates bladder epithelial damage (37).

In the epithelial injury hypothesis of IC, disruption of tight junctions has long been recognized as a pathogenic factor. Zonula occludens-1 (ZO-1) is a key tight junction protein responsible for maintaining the barrier function of the urothelium, preventing toxic metabolites and ions in the urine from penetrating below the mucosal layer of the bladder (29).

VEGF can activate Src family protein tyrosine kinase-dependent pathways, leading to the phosphorylation and inactivation of ZO-1. Animal studies in rats have revealed that this process results in the disruption of tight junctions between bladder umbrella cells, allowing toxic substances and ions from urine to infiltrate the subepithelial layer, ultimately triggering tissue damage (such as ulcers and punctate bleeding) and pain responses (29, 38, 39). Weis et al. demonstrated through animal experiments that following the activation of the VEGF signaling pathway, the expression of tyrosine kinases within this pathway was upregulated, promoting the phosphorylation of ZO-1 and mediating the breakdown of cell junctions. In tyrosine kinase knockout mice subjected to VEGF intervention, the phosphorylation levels of ZO-1 were significantly lower than those in normal mice, resulting in decreased vascular permeability and less severe tissue damage (p < 0.05) (40).

### 3.3 Epithelial repair

#### 3.3.1 PI3K-Akt/mTOR signaling pathway

**Figure 3:**
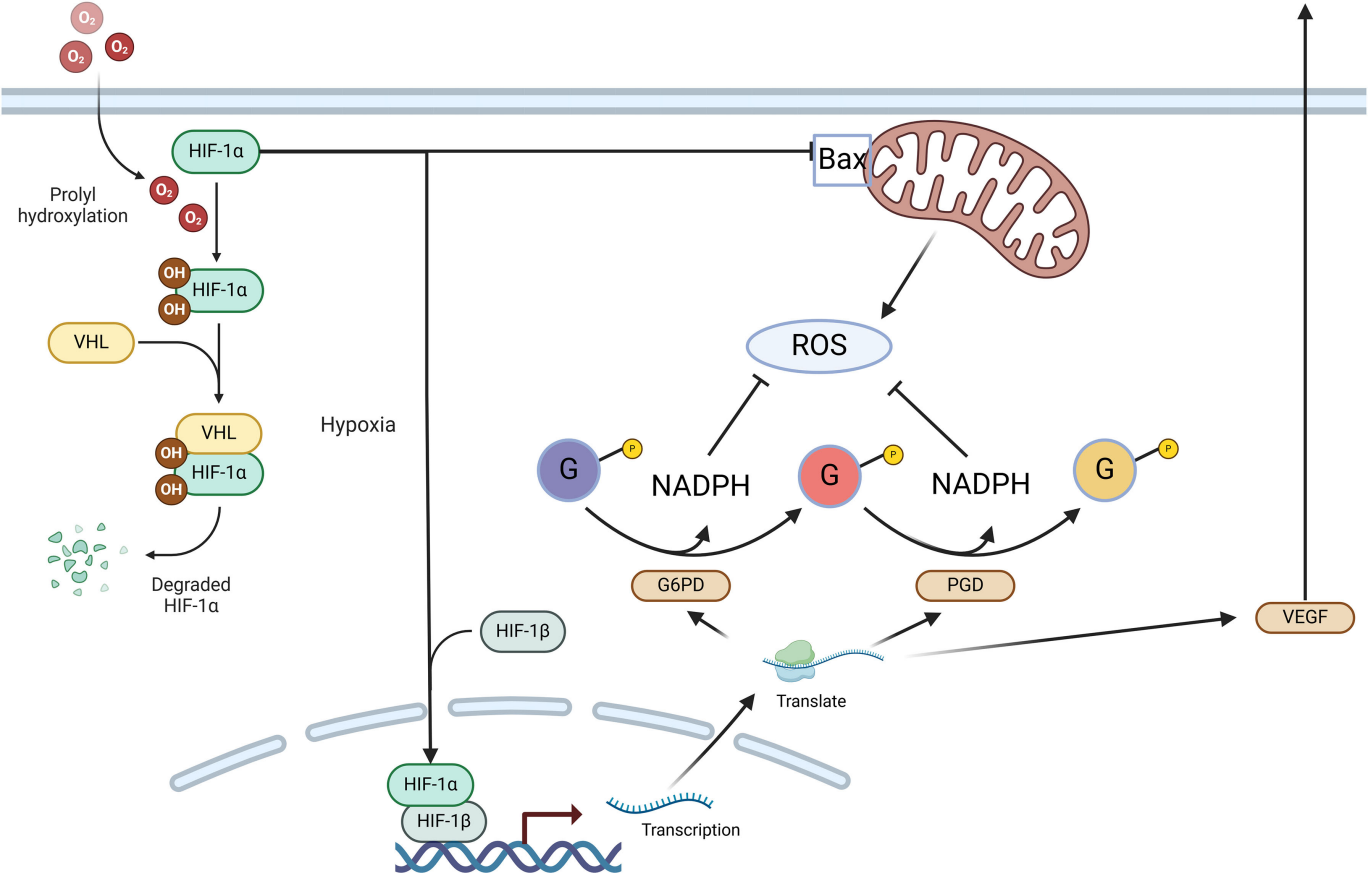
The diagram depicts the HIF-1 signaling pathway and its critical role in oxygen regulation and tissue protection, particularly in conditions like interstitial cystitis (IC). Under normoxia, HIF-1α undergoes hydroxylation, ubiquitination via VHL, and subsequent degradation. In hypoxic conditions, HIF-1α stabilizes, translocates to the nucleus, and pairs with HIF-1β to activate target genes such as VEGF, promoting angiogenesis and cellular survival. In IC, chronic bladder ischemia and hypoxia result in elevated HIF-1α expression, especially in ulcerated regions, contributing to capillary regeneration, reducing oxidative stress, and preventing hypoxia-induced apoptosis and epithelial injury. This pathway is crucial for mitigating bladder tissue damage seen in IC.

The phosphoinositide 3-kinase (PI3K) pathway is activated by the binding of various growth factors to their receptors, such as epidermal growth factor (EGF) and its receptor (EGFR). Once activated, PI3K phosphorylates Akt, leading to a conformational change that activates Akt. This phosphorylation event subsequently activates or inhibits a series of downstream substrates, including pro-apoptotic proteins like Bad and caspase-9, thereby regulating cell proliferation, differentiation, and apoptosis. The downstream target of the PI3K-Akt pathway is the mammalian target of rapamycin (mTOR), with downstream transcription factors such as HIF-1α and NF-κB that stimulate cell proliferation, enhance tissue resistance to damage, and promote tissue repair (41). In interstitial cystitis (IC), the PI3K-Akt/mTOR pathway facilitates the proliferation and repair of damaged bladder urothelium while inhibiting apoptosis, potentially playing a critical role in bladder fibrosis (**Figure 3**).

Xie et al. established an epithelial injury model by introducing exogenous TNF-α to cultured human urothelial cells (SV-HUC-1) to simulate the cellular environment during tissue damage. They then co-cultured this system with human umbilical cord mesenchymal stem cells (UC-MSCs) to mimic the treatment of bladder urothelium after stem cell transplantation. Results indicated that the MSC/SV-HUC-1 group exhibited a significant increase in phosphorylated Akt (P-Akt) and phosphorylated mTOR (P-mTOR) expression compared to the control group (42). *In vivo* experiments involved injecting UC-MSCs via the tail vein into a cyclophosphamide (CYP)-induced interstitial cystitis rat model. A single injection of UC-MSCs was found to significantly extend the bladder contraction interval, indicating enhanced urinary function in the IC rat model. After seven days, the MSC group displayed fewer mast cells compared to the IC group, along with notably lower levels of inflammatory cytokines IL-1β, IL-6, and TNF-α. Compared to the IC group, the UC-MSC injection group displayed new vascular structures under microscopy, with significant reductions in submucosal edema and hemorrhage.

EGF was found to be highly expressed in UC-MSCs and acts as an agonist of the PI3K-Akt/mTOR pathway (43, 44), to establish that the reparative effects of UC-MSCs are primarily due to the upregulation of the PI3K-Akt/mTOR pathway, EGF siRNA was used to knock out the EGF gene. The results indicated that tissue repair, represented by neovascularization and reduction of edema, was significantly diminished [46]. This evidence demonstrates that stem cells promote cell proliferation, tissue repair, and inflammation alleviation through the PI3K-Akt/mTOR pathway, providing a theoretical foundation for further research into the treatment of IC. Liu et al. employed a cyclophosphamide-induced rat cystitis model and found that the activity of the N-methyl-d-aspartate receptor (NMDAR) in the spinal cord, particularly through NR1 subunit phosphorylation, plays a crucial role in regulating bladder hypertrophy and dysfunction. NMDAR upregulates the expression of type I collagen in the bladder by activating the PI3K/Akt signaling pathway. Meanwhile, the NMDAR inhibitor MK-801 significantly alleviates cystitis-induced bladder weight increase, fibrosis, and urinary frequency symptoms (45).

The aforementioned research indicates that the pathological characteristics resulting from alterations in the PI3K/Akt pathway may primarily manifest as dysfunction, mild inflammation, and reversible damage. Under such conditions, activating the PI3K/Akt pathway contributes to maintaining urothelial integrity, repairing minor injuries, and suppressing excessive fibrosis, thereby alleviating symptoms and improving function. This aligns with the findings of Liu et al. (which demonstrated that NMDAR upregulates collagen via the PI3K/Akt pathway) as well as the results showing that stem cells facilitate repair through this pathway (45). Conversely, severe and structural damage may exceed the repair capacity of the PI3K/Akt pathway, which primarily functions to promote proliferation and inhibit apoptosis.

Current experimental evidence, primarily derived from CYP-induced animal models and *in vitro* cell co-culture systems, suggests that the PI3K-Akt/mTOR signaling pathway may play a dual role in IC/BPS. On one hand, activation of this pathway (e.g., via stem cells) helps maintain urothelial integrity, promote injury repair, and inhibit inflammation, thereby improving voiding function in animal models. On the other hand, this pathway (e.g., when activated by NMDAR) may also participate in the process of bladder fibrosis. However, due to the lack of direct support from human studies, the potential of this pathway as a therapeutic target for IC/BPS requires further validation and clarification in future clinical research.

## 4 Signaling pathways related to immunity and inflammation

### 4.1 NLR signaling pathway

**Figure 4:**
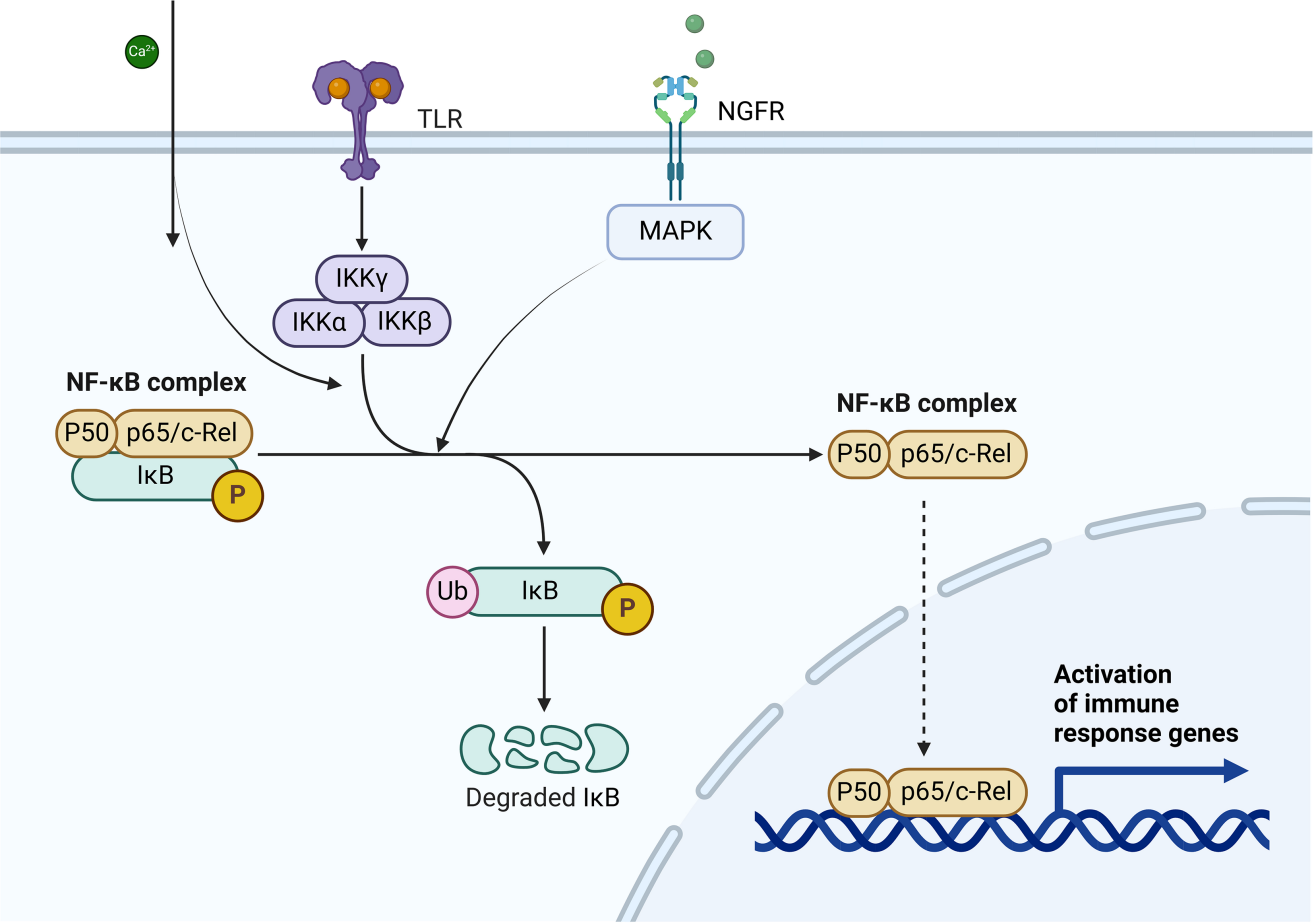
This figure illustrates the NF-κB signaling pathway, a critical pro-inflammatory mechanism with significant involvement in interstitial cystitis (IC). Toll-like receptor (TLR) activation leads to the recruitment of the IKK complex (IKKα, IKKβ, and IKKγ), which phosphorylates the inhibitory protein IκB. Phosphorylated IκB undergoes ubiquitination and proteasomal degradation, releasing the NF-κB complex (p50/p65). The liberated NF-κB translocates to the nucleus, where it activates the transcription of pro-inflammatory genes, including TNF-α and IL-1β. In interstitial cystitis, NF-κB activation in the spinal dorsal horn (SDH) and surrounding tissues upregulates TNF-α, IL-1β, and MAPK signaling markers such as p-p38 and p-JNK, leading to neuroinflammation. This neuroinflammation exacerbates pain hypersensitivity, lowers pain thresholds, and contributes to the chronic pain associated with IC. Furthermore, the persistent activation of NF-κB creates a self-amplifying inflammatory loop, perpetuating the release of cytokines and worsening symptoms in IC.

Composed primarily of nucleotide-binding and oligomerization domain-like receptors (NLRs), ASC proteins containing CARD, and the effector protein procaspase-1, the inflammasome is a multiprotein complex found in the cytoplasm. NLRP3 is the most well-known pattern recognition receptor in the NLR family and serves as a sensor for common pathogens and damage-associated factors. The NLRP3 inflammasome can be activated by infections or tissue damage; upon binding to pathogen products (e. g., bacterial RNA) or damage-associated factors (e. g., ROS, tissue cathepsin B), NLRP3 activates ASC, leading to the activation of procaspase-1 to caspase-1. Caspase-1 further processes the precursor of interleukin-1β (pro-IL-1β) to release mature IL-1, inducing pyroptosis (46–49). In IC, the activation of the NLR signaling pathway triggers the infiltration of inflammatory cells such as lymphocytes, neutrophils, and mast cells (50), resulting in bladder pain (51) and bladder fibrosis (**Figure 4**) (52).

Kiran et al. demonstrated that the blockade of NLRP3 could reduce the inflammatory response in IC. They employed the NLRP3 inhibitor DAP in a CYP-induced IC model in mice. The IC model group exhibited typical signs of bladder inflammation, including a significant increase in bladder wet weight and extensive infiltration of T cells, neutrophils, and mast cells; the treatment group showed reduced bladder wet weight and only minimal inflammatory cell infiltration compared to the model group (both p < 0. 05). Regarding immune cells, the treatment group exhibited a marked increase in T cells and dendritic cells (DCs) in the spleen and iliac lymph nodes (ILNs) compared to the IC model group, along with a significant reduction of CXCR3+ CD8+ T cells and DCs in the bladder (p < 0. 01). This indicates that during inflammation, DCs migrate from non-lymphoid to lymphoid tissues to activate naive T cells, which is a critical step in the inflammatory process; the chemokine receptor CXCR3 promotes T cell differentiation, inducing the production of effector and memory T cells, which are essential for the normal functions of T cells and mast cells. Furthermore, the NLRP3 blockade also alleviated the elevated expression of chemokines and inflammatory cytokines in IC model mice, including C-X-C motif chemokine ligand 10 (CXCL10), IL-1β, interferon-γ (IFN-γ), monocyte chemotactic protein-1 (MCP-1), and macrophage inflammatory protein-1α (MIP-1α) (p < 0. 05). These findings are consistent with previously observed results regarding inflammatory cell infiltration and align with the pathological conditions of IC patients. Therefore, it substantiates the critical role of NLRP3 in promoting the expression of inflammatory cytokines and chemokines, facilitating the proliferation and infiltration of inflammatory cells, and triggering inflammatory responses in IC (50).

The NLR signaling pathway acts as an activator of IL-1, playing a significant role in the initiation and progression of the inflammatory response in IC patients (53). Research by Wullt et al. elucidated the role of NLRP3 in inducing urinary frequency and pelvic pain in IC patients from the perspective of IL-1. The study included 17 patients, consisting of 7 BPS type 1A (NHIC) and 10 BPS type 3C (HIC) patients, who underwent comprehensive clinical evaluations and symptom scoring before and after treatment with IL-1 receptor antagonists. The results indicated that IL-1 receptor blockade improved urinary frequency (p = 0. 002), pelvic pain (p = 0. 002), and quality of life scores (p < 0. 001). Moreover, a whole-genome transcriptome analysis of tissue samples from IC patients was conducted, comparing expression levels during painful episodes and one week post IL-1 blockade, revealing that in 10 effective patients, 8 exhibited suppressed expression of IL-1 receptor genes (51). The upregulation of IL-1 is believed to trigger the inflammatory response in IC patients.

Additionally, the close association between the NLRP3 signaling pathway and tissue fibrosis has been validated in numerous animal models. Tissue fibrosis denotes a pathological process where chronic inflammation leads to parenchymal cell death, abnormal extracellular matrix buildup, and excessive proliferation of fibrous tissue (53). In chronic kidney disease, the knockout of the NLRP3 gene has been proven to protect against chronic kidney injury and reduce tissue fibrosis. Similarly, NLRP3 activation through IL-1 receptors on the bladder urothelium can induce epithelial-mesenchymal transition (EMT) and tissue fibrosis (52). After establishing a bladder fibrosis model, Francis et al. intervened with the NLRP3 blocker glibenclamide and the IL-1 blocker anakinra, resulting in significantly reduced bladder wet weight, collagen staining area, and collagen content compared to the control group (54). Shih et al. verified that NLRP3 regulates the TGF-β/Smad signaling pathway, leading to bladder fibrosis in IC. Following the instillation of lipopolysaccharide (LPS) and protamine sulfate (PS) into the bladders of normal mice to establish the IC model, model mice exhibited significantly increased urinary frequency (p = 0. 0001), markedly reduced bladder capacity (p = 0. 0065), and pronounced collagen Masson staining area and depth in the submucosa and muscle layer (p < 0. 0001); the expression levels of NLRP3 and IL-1β were significantly upregulated, alongside elevated expression of fibrosis markers such as TGF-β, Smad, vimentin, and E-cadherin. Following treatment with curcumin (an effective NLRP3 inhibitor), the bladder symptoms and tissue damage in mice were notably alleviated, with a significant reduction in fibrosis and decreased expression levels of IL-1, TGF-β, and Smad, confirming that NLRP3 mediates tissue fibrosis via the TGF-β pathway (55). Furthermore, EMT was observed in the urothelium and muscle layers, correlating with the histological features of urothelial shedding and subepithelial and smooth muscle fibrosis observed in IC (55). Wang et al. also demonstrated that the inhibition of NLRP3 inflammasome formation by pyrazole improved inflammatory necrosis of the urothelium in CYP model mice, further validating the role of this pathway in EMT (56).

Upon activation, the NLRP3 inflammasome strongly drives an inflammatory response centered on IL-1β, directly promoting the production of various pro-inflammatory cytokines (e.g., IL-1β, IL-6, TNF-α) and chemokines (e.g., CXCL10), which attract and activate extensive inflammatory cell infiltration. This aligns closely with the dense inflammatory cell infiltration observed in ulcerative interstitial cystitis (IC) (46–49). The clinical study by Wullt et al. clearly demonstrated that treatment with IL-1 receptor antagonists significantly improves urinary frequency, pelvic pain, and quality of life scores in patients with BPS type 3C (i.e., Hunner’s ulcer-type IC) (51). This directly proves that the NLRP3 inflammasome signaling pathway, by driving a strong IL-1β-centered inflammatory response, pyroptosis, and deep fibrosis, is highly consistent with the core clinicopathological features of Hunner’s ulcer-type IC, including severe inflammatory infiltration, tissue destruction, fibrosis, and intense pain. Clinical intervention studies also directly support the critical role of this pathway in ulcerative IC (51). Therefore, this pathway is more likely associated with Hunner’s ulcer-type IC and may represent a key pathogenic mechanism and therapeutic target.

### 4.2 P2X7 receptor-related signaling pathway

P2X receptors are purinergic signaling receptors that act as ligand-gated ion channels, with ATP as their primary ligand. P2X7, a subtype of P2X receptors, is distributed in both bladder smooth muscle and urothelium. Additionally, P2X7-induced K+ efflux is one of the initiating mechanisms for NLRP3 activation. The animal model study conducted by Negoro et al. using rats demonstrated that indicates that ubiquitin-binding protein-1 channels and P2X7 receptors are co-expressed in urothelial cells, and the binding of ATP to P2X7 receptors triggers the opening of the ubiquitin-binding pore (57). This results in ATP release, which can rapidly be metabolized into ADP, AMP, and adenosine, activating other purinergic receptors to accelerate smooth muscle relaxation or contraction (58–61). Martins et al. conducted experiments using wild-type mice, CYP model mice, CYP model mice treated with a selective P2X7 receptor antagonist, and P2X7 knockout CYP model mice. The results showed that P2X7 receptor expression was significantly elevated in CYP model mice, which also exhibited urinary frequency and heightened sensitivity to painful stimuli. The animals treated with antagonists and those in the knockout group displayed significantly reduced sensitivity to pain (p < 0. 01). Additionally, bladder tissue edema and hemorrhage, as well as the aggregation of macrophages and neutrophils, were significantly lower than in CYP model mice (p < 0. 05) (62). The P2X7 antagonist and knockout mice showed no significant variations in gene expression, tissue inflammation, or behavioral outcomes when compared to the CYP model receiving mesna, an agent that reduces side effects such as hemorrhagic cystitis induced by CYP. Therefore, the activation and upregulation of P2X7 channels are crucial contributors to urinary frequency, pelvic sensitivity, and tissue damage in IC. Moreover, P2X7 purinergic receptors participate in the inflammatory process of CYP-induced IC by increasing the migration of macrophages and neutrophils and producing pro-inflammatory cytokines.

The opening of mechanosensitive ion channels, specifically PIEZO channels, is one way the P2X signaling pathway can be activated. Once the PIEZO pathway is opened, the influx of Ca^2+^ triggers the release of ATP, which subsequently activates the P2X pathway that uses ATP as a ligand. In 2020, Kara L. Marshall et al. discovered that mechanosensitive ion channels are expressed in lower urinary tract tissues, functioning as sensors in bladder urothelium and sensory neurons, which are associated with the sensation of urgency and thereby linked to urinary frequency (63). Katharine et al. confirmed that in the CYP cystitis model, the expression of differentially expressed genes (Trpv1, Trpv4, PIEZO1, and PIEZO2) was upregulated in both urothelium and detrusor muscle. Furthermore, after intervention with the PIEZO activator Yoda1, the expression of tight junction genes (including Cldn1, Cldn8, and ZO-1) in the urothelium was downregulated, leading to increased bladder permeability. This indicates that the activation of PIEZO plays a role in urinary behavior, potentially representing a new therapeutic target for diseases such as IC/BPS (64).

The aforementioned studies demonstrate that the upregulation of P2X7 receptor activation directly mediates the sensitization of bladder sensory nerves, which is a key factor in causing core symptoms such as urinary frequency and pelvic pain. Meanwhile, the activation of PIEZO channels downregulates the expression of tight junction proteins, impairs urothelial barrier function, and increases bladder permeability. This provides a direct molecular mechanism for the core pathological hypothesis of non-Hunner lesion interstitial cystitis/bladder pain syndrome (NHIC-type IC/BPS)—the “leaky epithelium” theory (62). Furthermore, the infiltration of macrophages and neutrophils driven by this pathway represents a diffuse inflammatory response, which is consistent with the widespread bladder inflammation modeled by the cyclophosphamide-induced cystitis model used in the research, rather than the localized, severe necrotizing inflammation characteristic of Hunner ulcer-type IC/BPS (63). Therefore, through three main aspects—sensory dysfunction, epithelial barrier disruption, and diffuse inflammation—this pathway systematically explains the typical clinical manifestations and microscopic pathological changes observed in non-Hunner ulcer-type IC/BPS.

### 4.3 TGF-β signaling pathway

**Figure 5:**
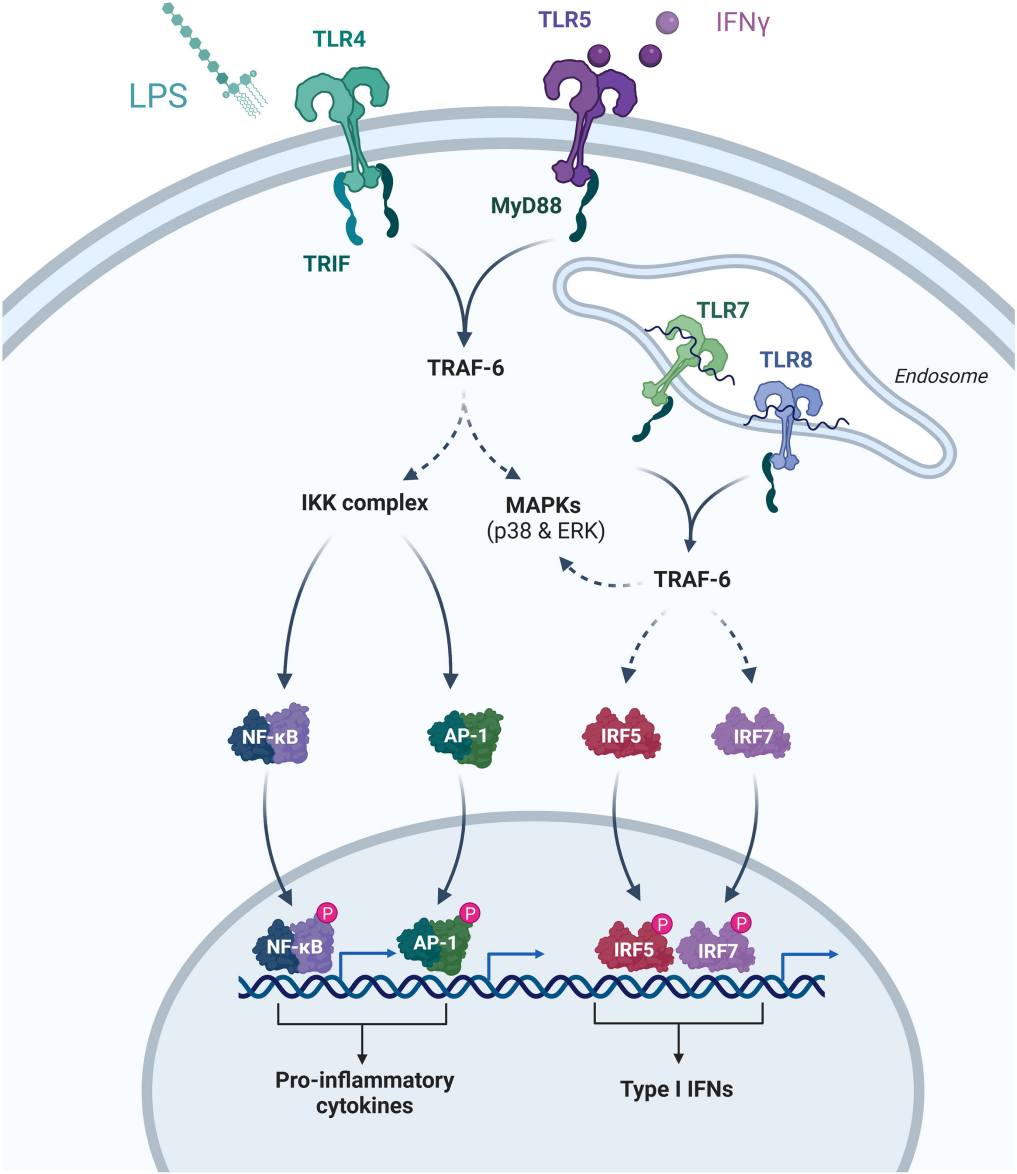
The diagram illustrates the Toll-Like Receptor (TLR) signaling pathway and its role in inflammation and pain in interstitial cystitis (IC). TLR4, activated by LPS, triggers MyD88- and TRIF-dependent pathways, leading to the activation of NF-κB, AP-1, and MAPKs (p38, ERK). These pathways result in the production of pro-inflammatory cytokines (IL-1β, IL-6, TNF-α). Endosomal TLR7/8, activated by IFNγ, signals through MyD88, inducing IRF5/7-mediated type I IFN production. In IC, upregulation of TLR4 and TLR7 correlates with inflammatory cytokine release, bladder pain, and central sensitization. TLR2 and TLR4 hyperactivity have been implicated in neuropathic pain pathways, linking TLR signaling to chronic pelvic pain and immune-mediated inflammation.

Transforming Growth Factor-β (TGF-β) plays diverse roles in cellular functions, including proliferation, apoptosis, differentiation, and migration. Upon binding to the type II receptor, TGF-β recruits the type I receptor, phosphorylating and activating it. Once phosphorylated, the type I receptor activates Smad proteins, which then form a complex that functions as a transcription factor (65). The TGF-β signaling pathway exhibits both pro-inflammatory and anti-inflammatory effects (66). However, research indicates that this cytokine primarily inhibits inflammation and promotes healing following damage to the upper urinary tract (67). In the tissue biopsies of IC patients, TGF-β1 expression is significantly elevated (68–71). TGF-β plays a dual role in IC; this pathway not only promotes the proliferation of epithelial cells and repairs tissue damage, alleviating pain and bladder irritation symptoms, but also participates in NLRP3-mediated epithelial-mesenchymal transition (EMT), leading to tissue fibrosis (**Figure 5**).

The regulation of inducible nitric oxide synthase (iNOS), responsible for nitric oxide (NO) production in inflammation, is strongly mediated by TGF-β1 (72). NO acts as a critical mediator and signaling molecule, supporting functions such as smooth muscle relaxation, neurotransmission, vasodilation, and urinary regulation (73). TGF-β1 substantially suppresses iNOS expression, thereby lowering NO levels within tissues (72). In IC patients, the bladder urothelium exhibits significant upregulation of both iNOS and NO (74). Tyagi et al. analyzed the levels of TGF-β and NO reaction products (NO2-/NO3-) in CYP-treated SD rats. Their findings revealed that, before CYP administration, female rats exhibited considerably higher urinary levels of NO2-/NO3- compared to males (p<0. 001), while urinary TGF-β1 levels showed no notable sex differences. Following the intraperitoneal injection of CYP, male rats showed a lower urinary NO2-/NO3- level in response to CYP compared to female rats, while urinary TGF-β1 levels significantly increased (p<0. 05), with the magnitude of increase far greater than that seen in female rats (p<0. 01). The levels of NO2-/NO3- were negatively correlated with the potential active form of TGF-β1. When NO2-/NO3- levels were at their lowest (24 hours post-CYP injection), urinary TGF-β1 levels peaked. These results indicate that TGF-β1 inhibits the generation of iNOS and NO, thereby alleviating inflammation caused by iNOS and its products. Furthermore, the significant sex-related differences in TGF-β and NO metabolites observed in CYP-induced IC could explain the higher prevalence of IC in female patients compared to male patients (75).

The binding of TGF-β to its receptors can activate the mitogen-activated protein kinase (MAPK) signaling pathway, which ultimately influences extracellular signal-regulated kinases (ERK), c-Jun N-terminal kinases (JNKs), and p38 through a cascade of kinase reactions, promoting cell proliferation and the development of inflammation (76). Xiao et al. investigated the role of the TGF-β/MAPK signaling pathway in urinary control and the effects on IC following the transplantation of bone marrow-derived mesenchymal stem cells. In their experiment, IC was simulated using bladder infusion of protamine sulfate. They found that in the stem cell implantation group, the expression of the TGF-β/MAPK signaling pathway was significantly upregulated compared to the model group. Furthermore, inflammatory cytokines (IL-2, IL-4, IL-10, TNF-α, and IFN-γ) were significantly reduced in the stem cell implantation group. Key functional improvements included increased maximum voiding volume, contraction intervals, and bladder capacity (P < 0. 05), while residual urine volume and bladder pressure were reduced (P < 0. 05). In terms of tissue morphology, the epithelial tissue in the stem cell implantation group remained largely intact, with less infiltration of neutrophils and mast cells, as well as reduced tissue edema compared to the model group (77). These findings are consistent with results from the MAPK inhibitor sp600125 treatment group, which also demonstrated reduced inflammatory cytokines, increased maximum voiding volume, contraction intervals, and bladder capacity, alongside decreased residual urine volume and bladder pressure. Therefore, inhibiting the TGF-β/MAPK signaling pathway has a positive effect on the control of urinary frequency and urgency symptoms, as well as on the repair of tissue damage in IC.

Mast cells are involved in innate and autoimmune responses, as well as neuroinflammatory diseases such as asthma, rheumatoid arthritis, and IC (78–80). The TGF-β pathway is closely related to mast cells; phosphorylation of the TGF-βI receptor on mast cells activates Smad proteins, which subsequently regulate gene transcription, leading to mast cell activation. Activated mast cells can increase cytokine expression and TGF-β production. Nobuhiro et al. demonstrated that TGF-β signaling promotes the proliferation and differentiation of mucosal mast cells (81), indicating a positive feedback loop between mast cells and TGF-β (82). Gamper et al. conducted bladder mucosal biopsies on 31 IC/BPS patients, revealing that the counts of subepithelial mast cells (p < 0. 001) and detrusor mast cells (p < 0. 029) were increased, which are characteristic features of IC (78). This further corroborates the positive correlation between the TGF-β signaling pathway and mast cells.

Additionally, TGF-β is involved in NLRP3-mediated epithelial-mesenchymal transition (EMT). Wang et al. isolated epithelial cells from Nlrp3-/- mice for their study. TGF-β downregulated the expression of matrix metalloproteinase (MMP) genes, which are widely recognized for their role in degrading extracellular matrix accumulation and alleviating fibrosis. In comparison to wild-type mice, the activity of TGF-β1-induced MMP-9 was significantly reduced in Nlrp3-/- mice (p < 0. 01). Moreover, Nlrp3 influenced the expression of TGF-β1-induced MMP-9 at the mRNA level. Following 24 hours of TGF-β1 stimulation, EMT morphological features were delayed in the Nlrp3-/- group. Compared to wild-type cells, the expression of epithelial cell markers E-cadherin and α-SMA was lower in the Nlrp3-/- group following TGF-β stimulation. These results indicate that Nlrp3 and TGF-β have a suppressive effect on MMP expression and promote EMT (83).

The core function of the TGF-β pathway highlights its role in tissue repair and anti-inflammation, such as promoting epithelial cell proliferation, alleviating pain and bladder irritation symptoms, and inhibiting iNOS/NO-mediated inflammation (68–71). These functions represent a response to diffuse, non-specific injury aimed at maintaining tissue homeostasis, rather than driving the formation of localized, severe ulcerative lesions. Although this pathway is also involved in fibrosis, this is a potential outcome under chronic or imbalanced conditions (78–80). In researching this pathway, the cyclophosphamide (CYP)-induced cystitis model and the protamine sulfate bladder infusion model are chemical, diffuse bladder injury models that effectively simulate the widespread inflammation, pain, and voiding dysfunction characteristic of IC/BPS (especially the non-Hunner lesion type), but they do not specifically produce Hunner’s ulcers (77). The intervention targets and observed efficacy endpoints in studies focused on this pathway are primarily centered on improving urinary frequency, urgency, bladder sensory function, tissue edema, and widespread inflammation—all of which are core therapeutic targets and clinical manifestations of non-Hunner lesion IC.

### 4.4 Wnt signaling pathway

**Figure 6:**
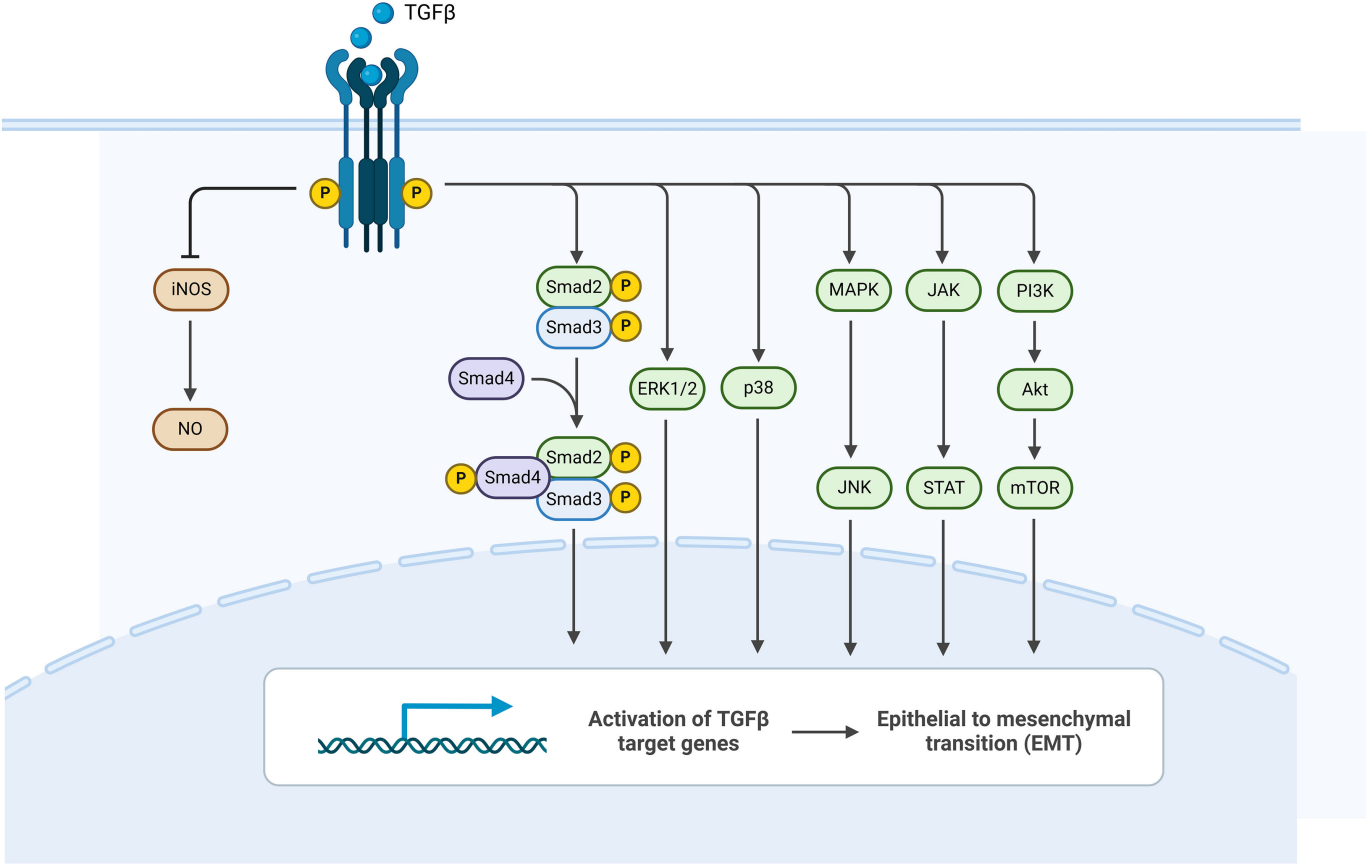
The diagram illustrates the TGF-β signaling pathway and its role in epithelial-mesenchymal transition (EMT) and inflammation in interstitial cystitis (IC). TGF-β binds to its receptors, phosphorylating Smad2/3, which forms a complex with Smad4 to activate downstream target genes, leading to EMT. Non-canonical pathways, such as MAPK (ERK1/2, p38, JNK), JAK/STAT, and PI3K/Akt/mTOR, are also activated, promoting cell proliferation, inflammation, and tissue repair. In IC, upregulation of TGF-β contributes to bladder epithelial damage, mast cell activation, and fibrosis. TGF-β also suppresses iNOS and NO production, reducing inflammation, while its activation promotes tissue remodeling and chronic bladder dysfunction through EMT processes.

The Wnt pathway is essential for ensuring the stability and homeostasis of urothelial cells. When the urothelium is damaged, Wnt can activate stem cell proliferation and differentiation at the basal layer, leading to rapid epithelial cell proliferation and subsequent tissue repair (84–86). In experiments conducted by Song et al., human UCB-MSCs were directly injected into the submucosal layer of the bladder in a CYP model rat, activating the Wnt signaling pathway. The results showed that the UCB-MSC group significantly increased the interval between bladder contractions and improved bladder voiding function (p < 0. 05), while new capillary formation and epithelial shedding were markedly reduced (p < 0. 05). Although there was some improvement in fibrosis, it was not statistically significant. Blocking the Wnt pathway with the inhibitor indomethacin resulted in a marked reduction in the recovery of bladder voiding function in the IC + MSC group, significantly affecting the regeneration of the epithelial layer. Cells stained for non-phosphorylated β-catenin, an indicator of Wnt activation, showed a marked reduction in number (p < 0. 001). These results indicate that the Wnt signaling pathway mediates the critical role of UCB-MSCs in the regeneration of urothelium in IC (**Figure 6**) (87).

Furthermore, the Wnt signaling pathway can promote tissue fibrosis and the associated epithelial-mesenchymal transition (EMT) process. The classical Wnt pathway facilitates EMT through mechanisms that include upregulation of mesenchymal markers like SLUG, ZEB1, and TWIST by Wnt/β-catenin, suppressing E-cadherin and increasing matrix metalloproteinases (MMPs) such as MMP-3, MMP-7, and MMP-9, resulting in matrix remodeling (88–91). However, Daeheon et al. found that the expression of Wnt genes in the bladder tissues of patients with non-Hunner’s interstitial cystitis (NHIC) was lower than that in patients with Hunner’s interstitial cystitis (HIC) (p < 0. 05). Notably, the expression of Wnt11 (a member of the Wnt family) was significantly suppressed in NHIC patients (p < 0. 05). Following the knockout of the Wnt11 gene, fibrosis was clearly observed under the microscope, and similar fibrotic changes were noted upon knocking out other target genes (WNT2B, WNT5A, and WNT10A). Daeheon et al. suggested that the silencing of Wnt genes activates the TGF-β signaling pathway and promotes epithelial-mesenchymal transition, leading to increased expression of fibronectin, which is a primary cause of bladder fibrosis in IC (92).

The above studies indicate that the Wnt signaling pathway plays different roles in different subtypes of IC (84–86).In Hunner-type Interstitial Cystitis (HIC), its expression is relatively high, primarily functioning to promote epithelial regeneration and repair tissue defects, which may be a positive response of the body to localized severe damage (88). In contrast, in Non-Hunner-type Interstitial Cystitis (NHIC), the silencing of Wnt genes (particularly Wnt11) instead becomes a key mechanism leading to bladder fibrosis by activating the TGF-β pathway (92).

### 4.5 Chemokine signaling pathway

Chemokines are critical molecules responsible for guiding inflammatory cells to sites of tissue damage or infection. During events like mechanical injury, infection, or inflammation, chemokines become highly expressed in diverse cell types, including neurons, glial cells, macrophages, T cells, and urothelial cells. Their main function involves creating a chemotactic gradient to direct cell migration toward higher concentrations, aiding in tissue repair and immune responses. Chemokines significantly influence tissue growth and restoration, promoting new blood vessel formation and supporting cellular proliferation and maturation. They also play a key role in leukocyte recruitment, drawing immune cells such as monocytes and neutrophils to affected areas and activating these cells for immune response initiation. In individuals with interstitial cystitis/bladder pain syndrome (IC/BPS), chemokine levels—including ligands like CCL2, CCL5, CXCL1, CXCL8, CXCL10, CXCR4, and CXCR3—are notably elevated in blood, urine, and bladder tissues compared to those without the condition (93–95). Similarly, animal models of cyclophosphamide-induced cystitis show heightened expression of chemokines, including CXCL12/CXCR4, CCL2/CCR2, and CX3CL1/CX3CR1, compared to controls (96). Zhao et al. investigated an experimental autoimmune cystitis (EAC) model by injecting mice with bladder homogenate supernatant. These mice exhibited similar symptoms to IC/BPS, such as bladder tissue swelling, inflammatory infiltration, and epithelial damage. Western blot and immunohistochemistry analyses revealed significant upregulation of CXCL13 and CXCR5 in this model (P < 0. 001). Using the CCR5 antagonist TAK-779, researchers observed substantial reductions in bladder swelling, inflammatory cell infiltration, and epithelial damage compared to untreated models (P < 0. 001). Additionally, levels of inflammatory mediators such as IL-6 (P = 0. 002), IL-1β (P = 0. 01), and TNF-α (P = 0. 008) were significantly reduced in treated tissues. Chemokines bind to their receptors and activate signaling pathways, including MAPK, PI3K, and NF-κB, which are crucial in managing inflammation, cell damage, and apoptosis, thereby contributing to the progression of IC pathology (96). The binding of chemokines to their receptors can activate various signaling pathways, including MAPK, PI3K, and NF-κB, which are involved in the regulation of tissue damage, inflammation, and apoptosis. These pathways play crucial roles in the pathological development of IC (97–100).

In the experimental autoimmune cystitis (EAC) model, mice exhibited diffuse pathological features highly consistent with non-Hunner lesion IC, such as bladder tissue swelling, inflammatory infiltration, and epithelial damage, along with significantly upregulated expression of CXCL13/CXCR5. Following treatment with the CCR5 antagonist TAK-779, these diffuse inflammatory changes and tissue damage were markedly alleviated, accompanied by a significant decrease in the levels of inflammatory mediators including IL-6, IL-1β, and TNF-α (96). In the cyclophosphamide (CYP)-induced diffuse cystitis model, the expression of multiple chemokines (CXCL12/CXCR4, CCL2/CCR2, CX3CL1/CX3CR1) was significantly elevated, further confirming the widespread activation of this pathway under diffuse inflammatory conditions (93–95). More importantly, clinical samples have demonstrated that levels of various chemokines in the blood, urine, and bladder tissues of IC/BPS patients (particularly those with the non-Hunner lesion type) are significantly higher than those in healthy individuals, establishing a direct association between this pathway and the human disease state. Mechanistically, chemokines participate in the regulation of inflammation, tissue damage, and apoptosis by activating downstream signaling pathways such as MAPK, PI3K, and NF-κB—precisely the core molecular mechanisms that drive neurogenic inflammation and immune activation in non-Hunner lesion IC (97–100).

### 4.6 JAK-STAT signaling pathway

Janus kinase 3 (JAK3) is a non-receptor tyrosine kinase (101). JAK3 plays a role in interleukin and cytokine signaling pathways by phosphorylating downstream STAT proteins. The Janus kinase (JAK)/signal transducer and activator of transcription (STAT) pathway is involved in critical cellular processes such as survival, proliferation, and inflammation. MicroRNA-132 acts as a downstream effector of the JAK-STAT signaling pathway, regulating the expression of JAK-STAT components and thereby influencing signal transduction (102). Song et al. validated the pro-inflammatory and fibrotic roles of the JAK-STAT pathway in interstitial cystitis (IC) by inhibiting the biological function of MicroRNA-132 in a rat model of interstitial cystitis. The results indicated that the MicroRNA-132 inhibition group showed minimal neutrophil and lymphocyte infiltration, with a reduction in mast cells. Collagen fibers in the tissue were significantly decreased, and the expression of collagen types I and III was notably reduced. Additionally, bladder capacity increased, and urinary frequency decreased (P < 0. 05), with significant reductions in the levels of inflammatory molecules IL-6, IL-10, IFN-γ, TNF-α, and ICAM-1 (P < 0. 05) (103). Conversely, Hou et al. investigated the role of MicroRNA-495 in alleviating tissue inflammation in IC by inhibiting JAK3, thereby inactivating the JAK-STAT signaling pathway. They found that MicroRNA-495 was downregulated in IC rats while JAK3 was upregulated. Following treatment with MicroRNA-495, the bladder wet weight and wet weight-to-body weight ratio of the IC model rats significantly decreased, and the degree of tissue fibrosis was notably reduced (P < 0. 05). qPCR and Western blot analyses demonstrated that after overexpression of MicroRNA-495, JAK3 expression decreased significantly. Conversely, blocking MicroRNA-495 in the intervention group led to upregulation of JAK3, which exacerbated bladder tissue inflammation and fibrosis (104). These two sets of experiments provide evidence from both positive and negative perspectives, confirming the pro-inflammatory and pro-fibrotic roles of the JAK-STAT pathway in IC.

In animal experiments, inhibition of the JAK-STAT pathway resulted in reduced diffuse infiltration of neutrophils, lymphocytes, and mast cells, decreased collagen fiber deposition and interstitial fibrosis, as well as improvements in functional voiding symptoms such as increased bladder capacity and reduced urinary frequency—findings that represent the core clinical manifestations of NHIC patients. From a molecular mechanism perspective, MicroRNA-132 and MicroRNA-495 regulate the JAK-STAT pathway, influencing the expression of various inflammatory molecules including IL-6 and TNF-α, as well as collagen expression. This regulation of the extensive inflammatory and fibrotic network perfectly aligns with the complex pathophysiological processes of NHIC (102). Furthermore, the models employed in the research, such as the CYP-induced diffuse cystitis model, exhibit characteristics of diffuse inflammation and dysfunction that are widely recognized as primarily simulating non-Hunner lesion IC/BPS (103). Therefore, the JAK-STAT signaling pathway is more inclined to explain the pathogenesis of non-Hunner lesion interstitial cystitis.

### 4.7 Toll-like receptor signaling pathway

**Figure 7:**
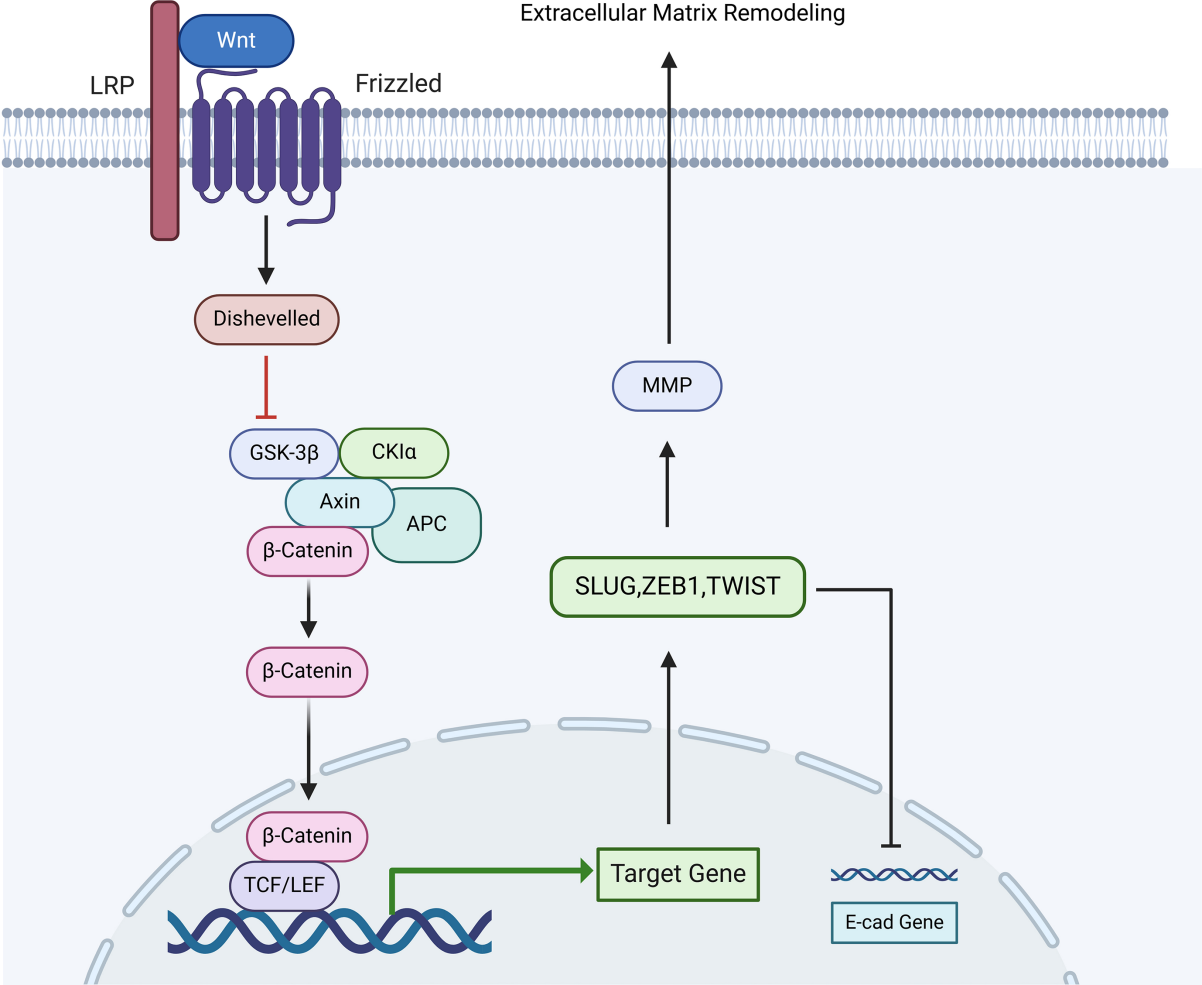
The diagram illustrates the Wnt/β-catenin signaling pathway and its involvement in tissue repair and epithelial-mesenchymal transition (EMT) in interstitial cystitis (IC). Wnt binds to Frizzled and LRP receptors, activating Dishevelled, which inhibits GSK-3β, stabilizing β-catenin. β-catenin translocates to the nucleus, activating target genes via TCF/LEF, leading to epithelial cell proliferation and tissue repair. Additionally, Wnt signaling induces EMT by upregulating mesenchymal markers (SLUG, ZEB1, TWIST) and promoting matrix metalloproteinase (MMP) expression, suppressing epithelial markers like E-cadherin. In IC, Wnt pathway dysfunction or gene silencing can trigger fibrosis through TGF-β activation, exacerbating bladder tissue damage and chronic inflammation.

Toll-like receptors (TLRs) are immune cell membrane glycoproteins that bind pathogen-derived molecules to initiate inflammatory signaling cascades. TLR signaling pathways are divided into two groups: (1) the MyD88-dependent pathway, which produces pro-inflammatory cytokines (such as IL-1, IL-6, TNF-α) through the rapid activation of NF-κB and MAPK; (2) the MyD88-independent pathway, activated by IFN-β and interferon-inducible genes, associated with dendritic cell maturation and the slower activation of NF-κB and MAPK (105–107). Both signaling pathways result in the expression and release of inflammatory cytokines, leading to an inflammatory response (**Figure 7**).

In bladder biopsies from patients with HIC, an upregulation of TLR7 expression has been observed. Additionally, increased levels of TLR7 ligands lead to elevated TLR7 mRNA expression, inducing cystitis in mice. Research has also shown that TLR4 is expressed on urothelial cells and rapidly activates inflammatory cytokines during bladder infectious diseases, mediating the inflammatory response (108–111). Andrew et al. demonstrated a correlation between TLR-4 inflammatory scores and measurements of non-specific pain intensity and frequency. Notably, the composite TLR-4 inflammatory score (calculated based on IL-6 and IL-1β responses) showed significant correlations with higher scores on the Genitourinary Pain Index (GUPI) total score (p = 0. 005) and GUPI pain subscale score (p = 0. 010). Specifically, pain frequency (p = 0. 001) and intensity (p = 0. 008) had the most significant associations with TLR-4 inflammatory scores. However, no correlation was found between TLR-4 inflammatory scores and urinary urgency, frequency, or nocturia in the Interstitial Cystitis Symptom Index (ICSI) (p > 0. 17), nor with bladder-specific pain/burning symptoms (p = 0. 13). Furthermore, TLR-2 hyperreactivity was associated with early or widespread features of IC/BPS, unlike TLR-4 sensitivity. Notably, IFN levels only significantly increased following TLR-4 stimulation (109). Kwok et al. investigated enhanced TLR ligand responsiveness in patients with chronic pain. They found that stimulation of either TLR-2 or TLR-4 resulted in increased activity of the NF-κB family. However, only when TLR-2 was stimulated did the IL-1β response increase sufficiently to differentiate chronic pain patients from healthy controls in peripheral blood mononuclear cells, suggesting that TLR-2 could be a potential diagnostic marker for IC in the future (112).

Endogenous pain signals (such as IL-1β, TNF-α, IL-6, and NO) activate TLRs. TLR2 on astrocytes and TLR2, TLR3, and TLR4 on microglia mediate neuropathic pain. Studies using TLR2 knockout mice demonstrated that after inducing nerve injury, these animals exhibited significantly lower thresholds for mechanical and thermal stimuli compared to wild-type controls, with a corresponding decrease in IL and TNF-α secretion (113). Similarly, antagonizing TLR4 in the spinal cord was shown to reverse neuropathic pain caused by sciatic nerve injury; the application of inhibitors like naloxone significantly lowered mechanical and thermal pain thresholds and downregulated related inflammatory molecules in the injured animals (114). This aligns with the characteristic of IC patients, where pelvic pain leads to central sensitization and subsequently manifests as somatic symptoms.

A series of studies have demonstrated that the Toll-like receptor signaling pathway is confirmed to be more inclined to explain the pathological mechanism of Hunner-type interstitial cystitis (HIC). The most direct evidence comes from bladder tissue biopsies of HIC patients, showing a significant upregulation of TLR7 expression, and increased levels of its ligand can directly induce cystitis in mice, linking the abnormal expression of TLR7 specifically to the HIC subtype. Clinical correlation studies further revealed that the TLR-4 inflammation score (based on IL-6/IL-1β responses) is significantly associated with pain frequency and intensity, but shows no correlation with urinary symptoms such as urgency and frequency—a finding that perfectly aligns with the clinical characteristics of HIC patients, where pain is prominent while urinary symptoms are relatively secondary (108–111).

## 5 Signaling pathways related to neuroinflammation and central sensitization

### 5.1 VEGF signaling pathway

VEGF plays a pivotal role in central sensitization related to bladder pain symptoms in IC/BPS. Neuropilin (NRP), an alternative receptor for VEGF, influences the directional growth of axons and organ innervation (115). The interaction between VEGF and NRP guides concurrent growth of blood vessels and nerves (116). NRPs are highly expressed in the bladder urothelium and intramural ganglia of mice, providing a structural foundation for bladder pain conduction. Additionally, NRP expression is significantly upregulated in BCG-induced cystitis (30) and cyclophosphamide-induced cystitis (117) (both p<0. 05). NRP is also co-localized with integrins in mouse and human bladder urothelium, forming an NRP-integrin complex that modulates function in response to VEGF stimulation (118). Marcia R. and colleagues quantified changes in peripheral neural plasticity using the density of transient receptor potential vanilloid subfamily 1 (TRPV1) and the neuronal marker PGP9. 5. They found that BCG instillation induced a marked inflammatory response, characterized by suburothelial infiltration of inflammatory cells and neovascularization, alongside a notable increase in the immunoreactivity of PGP9. 5 and TRPV1 in the bladder suburothelium, revealing a significant increase in peripheral nerves associated with IC. Treatment with an anti-VEGF neutralizing antibody (B20) attenuated the effects of BCG on inflammation and nerve density, resulting in symptoms of increased urinary frequency and pelvic sensitivity. Additionally, treatment with anti-VEGF (B20) and anti-NRP antibodies in mice reduced BCG-induced tissue inflammation levels and peripheral nerve density (p<0. 05). These findings indicate that VEGF, through its interaction with NRP, increases the suburothelial peripheral nerve population, leading to enhanced sensory afferent input, which directly heightens neural responses in the bladder under inflammatory conditions and triggers bladder region pain. This evidence highlights VEGF’s role in promoting concurrent neurovascular growth (119).

### 5.2 PI3K-mTOR signaling pathway

Stimulation of nociceptors in sensory nerves triggers the release of phospho-L-aspartate and calcitonin gene-related peptide (CGRP) from nerve terminals in the dorsal horn’s superficial layers, leading to nociceptive impulses that elicit bladder pain. CGRP receptors, located presynaptically on nerve terminals, facilitate the binding of substance P and CGRP to their respective receptors, resulting in the sensation of pain. The PI3K-mTOR pathway can indirectly influence CGRP receptors, thereby reducing substance P and CGRP levels to alleviate pain. The dorsal horn interneurons with active PI3K-mTOR pathways may be another source of substance P and CGRP. Elevated expressions of p-mTOR, mTOR-p-S6K1, p-4E-BP1, and p-PI3K in CYP rats suggest their involvement in IC-induced pain. Intrathecal injection of mTOR inhibitor (rapamycin) or blocking PI3K upstream of mTOR reduces bladder hypersensitivity and pain (120–122). Additionally, post-inhibition of mTOR or PI3K results in a marked decrease in CYP-induced spinal expression levels of substance P and CGRP (P<0. 05).

Liang et al. found that PI3K/mTOR signaling contributes to bladder hypersensitivity and pain in CYP rats. Treatment with mTOR inhibitor rapamycin and PI3K inhibitor LY294002 revealed increased p-mTOR, p-S6K1, and p-4E-BP1 in the dorsal horn. Administering rapamycin into the dorsal horn alleviated mechanical hyperalgesia, restored CYP-induced reductions in contraction intervals, and decreased pain sensitivity in CYP rats (p<0. 05), indicating that PI3K/mTOR signaling mediates CYP-induced bladder mechanical pain and hypersensitivity (123).

Liu et al. revealed that activation of Akt mediated by N-methyl-D-aspartate receptor (NMDAR) could be a mechanism underlying bladder hyperactivity and pain. NMDAR, an ionotropic glutamate receptor, is well-documented to alter synaptic excitation/inhibition thresholds. Following 48 hours of cyclophosphamide-induced cystitis, NMDAR activity—measured via phosphorylation levels of the NMDAR 1 subunit—was expressed in the spinal cord but absent in the bladder. Inhibition of NMDAR with dizocilpine (MK-801) significantly reduced bladder weight increase and collagen type I upregulation in the bladder, and behavioral experiments in CYP model rats showed decreased voiding frequency and reduced bladder smooth muscle sensitivity (45). These findings indicate that Akt activation in spinal cord cells contributes to bladder pain.

### 5.3 Neurotrophic factor-TNF-α/NF-κB pathway

**Figure 8:**
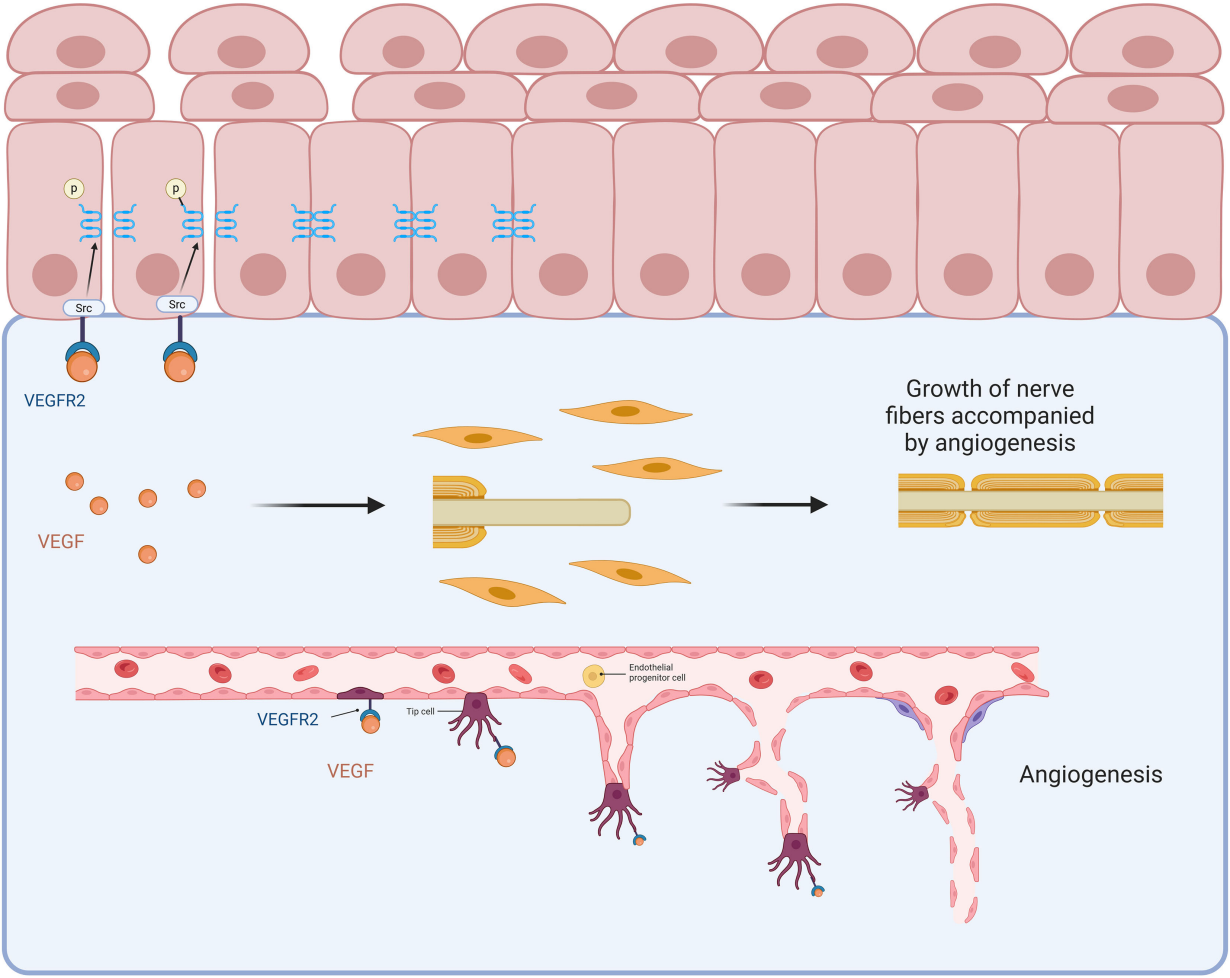
The diagram highlights the role of the VEGF signaling pathway in angiogenesis and epithelial injury, particularly in the context of interstitial cystitis (IC). VEGF binds to VEGFR2 on the bladder urothelial cells, activating downstream pathways such as Src kinase, which phosphorylates junctional proteins like ZO-1. This disrupts tight junction integrity, increasing urothelial permeability and allowing toxic substances to penetrate the subepithelial layers, contributing to epithelial damage and pain. VEGF-induced angiogenesis promotes new capillary formation but results in fragile vasculature, exacerbating petechial bleeding observed in IC. Additionally, VEGF stimulates nerve fiber growth, linking angiogenesis to bladder pain hypersensitivity.

Neurotrophic factors comprise a family of proteins that includes nerve growth factor (NGF), brain-derived neurotrophic factor (BDNF), neurotrophin-3 (NT-3), and neurotrophin-4 (NT-4). T These proteins function via Trk tyrosine kinase receptors or the p75 neurotrophin receptor (p75NTR) and are activated by pathways like MAPK, PI3K, and PLC. These signaling mechanisms support neural development, cell survival, and processes like learning and memory (**Figure 8**) (124–126).

The NF-κB signaling pathway is a critical pro-inflammatory pathway that can be activated by neurotrophic factors, with phosphorylated p65 (p-p65) serving as the primary transcriptional regulator of NF-κB pathway activation. TNF-α activates NF-κB by stimulating the constitutively expressed TNF receptor 1 (TNFR1) on sensory neurons, leading to the overexpression of pro-inflammatory cytokines and their receptors. Furthermore, TNF-α promotes p65 phosphorylation, a key step in the NF-κB pathway, driving its nuclear translocation and subsequent activation of target genes, including itself and IL-1β. This self-amplifying mechanism mediated by autocrine signaling can lead to aberrant pain behaviors through the continuous production of pro-inflammatory cytokines, predominantly TNF-α (127, 128).

In the spinal dorsal horn (SDH) of rats with IC, upregulation of Iba1 (a marker of microglial activation), GFAP (glial fibrillary acidic protein, a marker of astrocyte activation), p-p38 (a MAPK), p-JNK, IL-1β, and TNF-α has been observed. Activated astrocytes induce the upregulation of the MAPK pathway, promoting the synthesis and release of IL-1 and TNF-α, leading to neuroinflammation, decreased pain thresholds, and persistent pain. Astrocytes release IL-1, which induces the phosphorylation of NMDA receptors in SDH neurons (129).

Neuroinflammation in neuropathic pain is primarily driven by the TNF-α/NF-κB pathway and elevated IL-1β levels in the spinal cord. TNF-α-induced inflammation in the hippocampus might explain connections between pain, depression, and memory difficulties. Research using Western blot showed increased TNF-α, p-p65/p65, and IL-1β in SDH and hippocampus of cystitis rats. PDTC treatment reduced hyperalgesia and normalized depression-like behavior. (129–132).

### 5.4 JAK-STAT signaling pathway

The JAK/STAT pathway acts as a key signaling route for cytokines and growth factors. Cytokine binding to receptors activates STAT proteins through JAK kinases. Activated STATs form dimers and move to the nucleus to regulate gene expression. This pathway enhances bladder hyperreflexia and pain caused by CYP-induced inflammation (103). Song et al. administered the JAK2 inhibitor AG490 to rats treated with CYP and observed that the AG490 group exhibited minimal neutrophil and lymphocyte infiltration, with tissue conditions showing no significant differences compared to the wild-type group (p > 0. 05). In comparison to the IC group, there was a significant reduction in mast cell infiltration (p < 0. 05), along with increased bladder volume and prolonged bladder contraction intervals (p < 0. 05) (103). Cheppudira et al. further investigated the effects of AG490 on JAK/STAT inhibition in CYP-treated rats, measuring paw sensitivity using von Frey filaments. Their results indicated that the pain threshold in the inhibition group was significantly higher than that in the CYP model group (p ≤ 0. 05), confirming that JAK inhibition markedly reduced bladder hyperreflexia and hind paw sensitivity in rats. This indicates that the JAK/STAT pathway mediates pain and hyperalgesia in IC (133).

### 5.5 NOTCH signaling pathway

**Figure 9:**
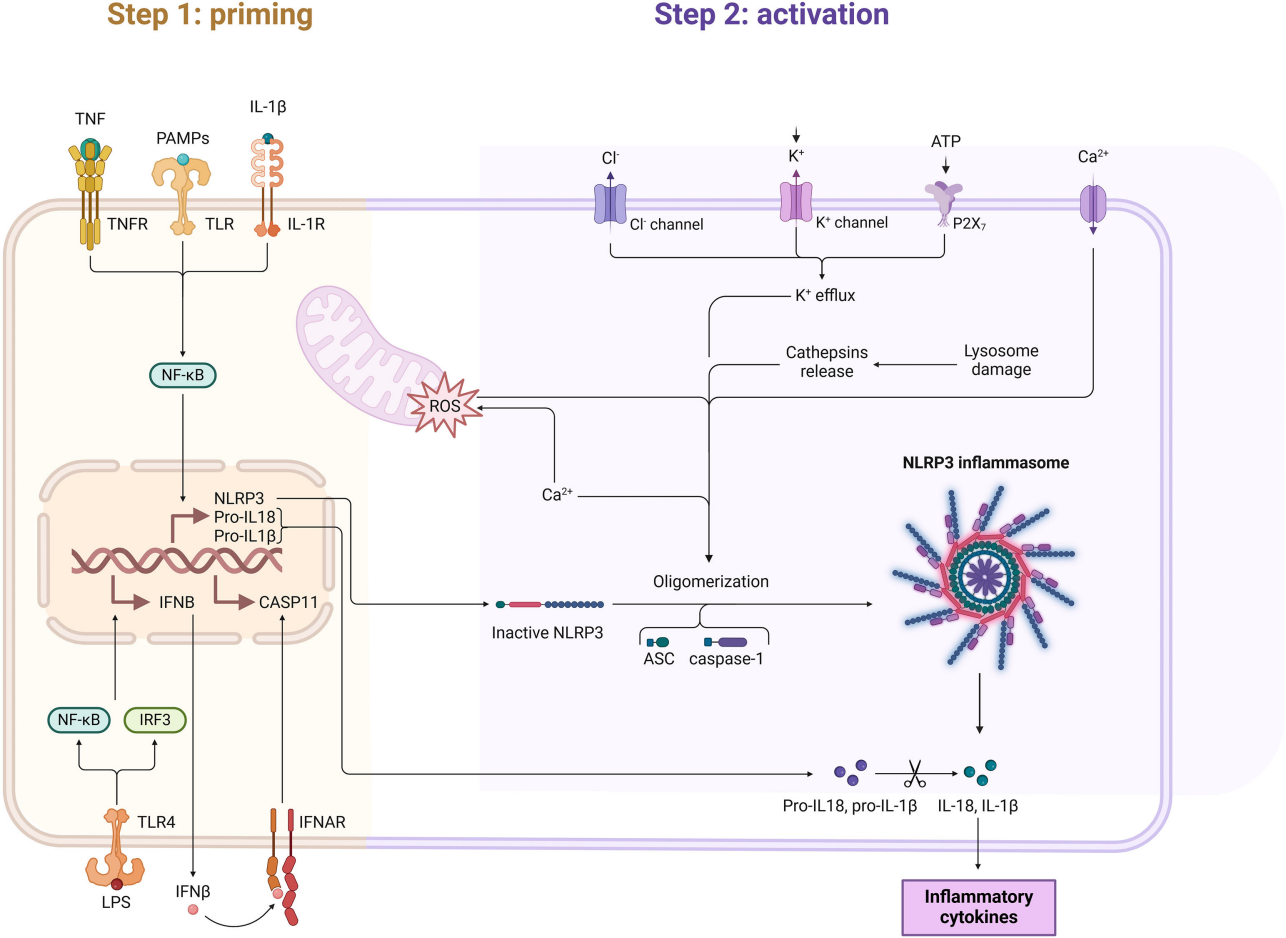
The diagram illustrates the NLRP3 inflammasome activation pathway and its role in inflammation and fibrosis in interstitial cystitis (IC). The process occurs in two steps: priming and activation. In step 1, priming begins with the activation of NF-κB via TNF, TLRs, or IL-1 receptors, leading to the transcription of NLRP3, pro-IL-1β, and pro-IL-18. In step 2, cellular stress signals, including ROS production, K^+^ efflux, lysosome damage, and Ca²^+^ influx, activate the NLRP3 inflammasome. Oligomerized NLRP3 activates caspase-1, cleaving pro-IL-1β and pro-IL-18 into active inflammatory cytokines, which induce pyroptosis and trigger inflammatory responses. In IC, this pathway promotes inflammatory cell infiltration, cytokine release, bladder pain, and tissue fibrosis.

Notch is a membrane receptor that, upon cleavage by gamma-secretase, releases the intracellular Notch receptor domain (NICD), which then translocates to the nucleus. Within the nucleus, NICD binds to transcription factors to regulate cell proliferation, differentiation, and apoptosis. Notch1 is upregulated in CYP-treated rats and promotes microglial activation and neuroinflammation, mediating mechanical allodynia associated with CYP-induced cystitis and decreasing pain thresholds, ultimately contributing to pelvic pain in interstitial cystitis/bladder pain syndrome (IC/BPS). Inhibition of Notch1 can alleviate pathologic pain caused by neuroinflammation (**Figure 9**).

Chen et al. measured the expression levels of Notch1 and NICD in the spinal dorsal horn (SDH) of the cystitis group in the CYP model, finding significant upregulation. They subsequently inhibited Notch1 signaling through intrathecal injection of the gamma-secretase inhibitor DAPT. Pain thresholds were assessed using von Frey filaments to evaluate bladder pain sensitivity, and results showed that continuous intrathecal injection of DAPT over three days significantly reversed the decrease in pain thresholds in cystitis animals after CYP induction (P < 0. 001). Western blotting (WB) and immunofluorescence staining revealed marked reductions in the activation markers Iba-1, p-p38, and OX-42 in the SDH (p < 0. 05), indicating that gamma-secretase inhibition reversed the excessive activation of microglia in the SDH of CYP rats. Additionally, the production of inflammatory cytokines such as TNF-α and IL-1β was similarly reduced following gamma-secretase treatment (p < 0. 05), alleviating the neuroinflammation induced by microglial activation, which correlates with the increased pain thresholds observed. This evidence supports a negative correlation between the expression of Notch1 and NICD and the mechanical sensitivity thresholds in cystitis rats. Therefore, inhibiting the Notch signaling pathway may have therapeutic potential for pelvic pain associated with BPS/IC (134).

## 6 Signal pathway crosstalk and the integrated network mechanism of IC/BPS

**Figure 10:**
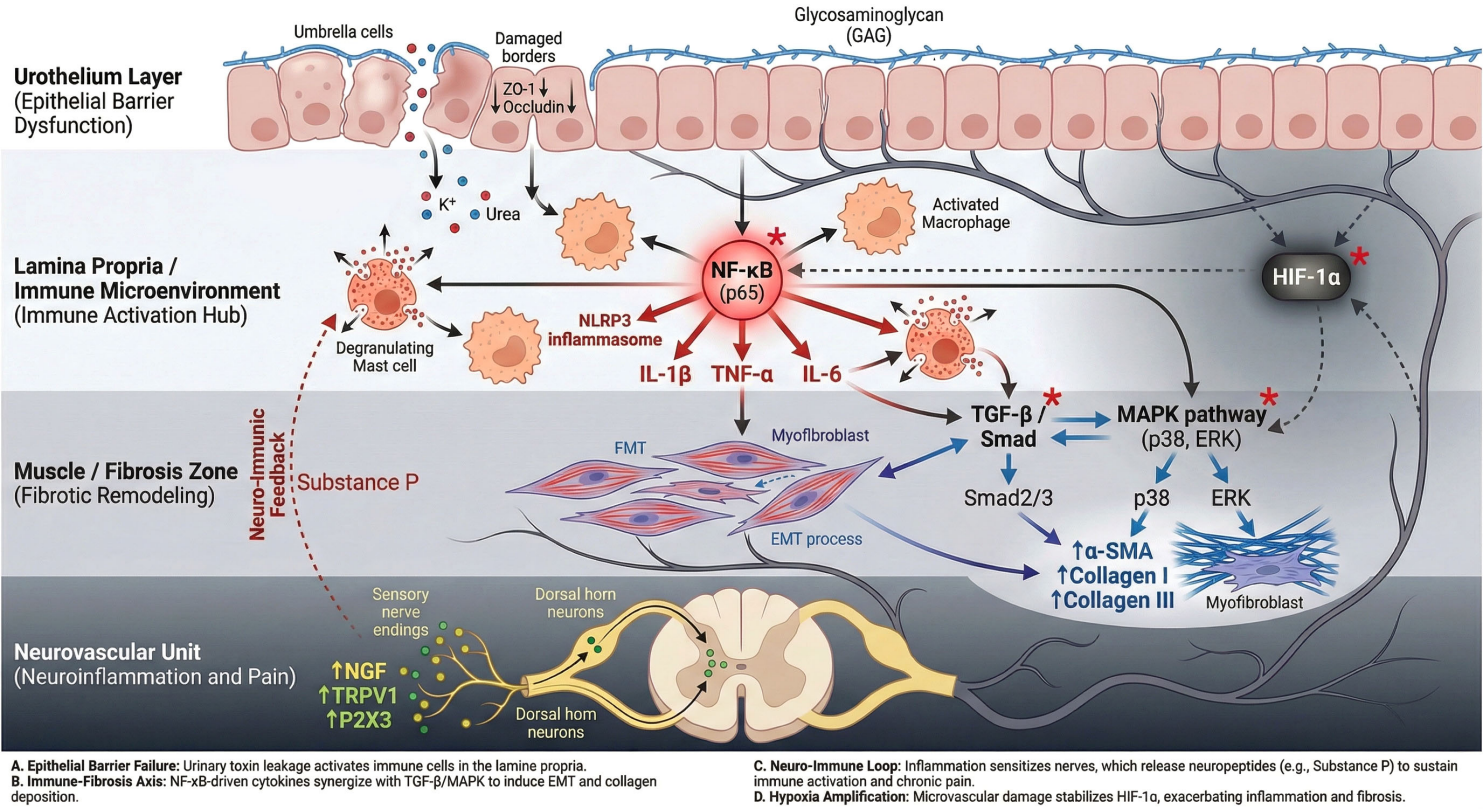
Signal pathway crosstalk and the integrated network mechanism of IC/BPS.

Although the preceding sections have elaborated on the independent roles of specific signaling pathways in Interstitial Cystitis/Bladder Pain Syndrome (IC/BPS), clinical and experimental evidence indicates that the pathological progression of IC/BPS is not a linear accumulation of single pathways. Instead, it constitutes a complex, dynamic network formed by the multi-system interaction of urothelial damage, immune activation, tissue fibrosis, neuroinflammation, and the hypoxic microenvironment. Constructing this unified disease network model is crucial for understanding the mechanisms of chronicity and for developing targeted therapies (**Figure 10**).

### 6.1 Cascade reaction of epithelial barrier dysfunction and immune initiation

The initiating factor of the disease often stems from damage to the urothelial barrier function. Downregulation of tight junction proteins (such as ZO-1, Occludin) and loss of the glycosaminoglycan (GAG) layer lead to the leakage of toxic urinary substances (e.g., potassium ions, urea) into the lamina propria. This chemical stimulus directly activates mast cells and macrophages in the lamina propria, triggering the nuclear translocation of the NF-κB signaling pathway. Activated NF-κB not only initiates the assembly of the NLRP3 inflammasome, promoting the maturation and release of IL-1β and IL-18, but also upregulates the transcription of pro-inflammatory factors like TNF-α and IL-6. These inflammatory mediators constitute the core driving force of immune activation and act as signaling molecules, transmitting signals downstream to the fibrotic and pain pathways.

### 6.2 Molecular crosstalk in the inflammation-fibrosis axis

The chronic inflammatory microenvironment is key to driving bladder wall fibrosis. Pro-inflammatory factors (particularly TNF-α and TGF-β1) activate fibroblasts via paracrine signaling. In this process, significant crosstalk occurs between the canonical TGF-β/Smad pathway and the non-canonical MAPK (ERK1/2, p38) pathways. ROS (Reactive Oxygen Species), acting as crucial second messengers, can be produced by inflammatory cells and oxidatively inhibit phosphatases, thereby enhancing the phosphorylation levels of MAPKs. This, in turn, synergizes with Smad proteins to promote the expression of α-SMA and Collagen I/III, inducing Epithelial-Mesenchymal Transition (EMT). This synergistic effect at the molecular level accelerates the excessive deposition of the extracellular matrix, leading to decreased bladder compliance.

### 6.3 Neuro-immune feedback loop and pain sensitization

Pain is not merely a symptom of the disease but also an active factor maintaining the pathological state. Inflammatory mediators (such as NGF, Prostaglandins) directly act on afferent nerve endings, upregulating the expression of TRPV1 and P2X3 receptors, lowering the pain threshold, and leading to peripheral sensitization. More critically, sensitized sensory nerve endings can retrogradely release neuropeptides (such as Substance P, CGRP), a phenomenon known as “neurogenic inflammation.” Released Substance P can directly bind to NK-1 receptors on the surface of mast cells, inducing their degranulation and the release of histamine and more cytokines, thus forming a “neuro-immune positive feedback loop.” This feedforward circuit explains why pain and inflammation can persist even after the initial injury has healed.

### 6.4 Amplifying effect of the hypoxic microenvironment

As fibrosis worsens and vascular endothelial injury occurs, microvascular density in the bladder wall decreases, leading to tissue hypoxia. Hypoxia-Inducible Factor-1α (HIF-1α) is stabilized under hypoxic conditions and acts as a global regulator, integrating the aforementioned network. HIF-1α not only promotes VEGF expression in an attempt to stimulate angiogenesis but also enhances the transcriptional activity of NF-κB, further exacerbating inflammation. Simultaneously, it can directly induce the expression of fibrosis-related genes. Hypoxia, inflammation, and fibrosis become mutually causative, forming a vicious cycle that makes the disease resistant to self-resolution.

In summary, NF-κB, MAPK, and TGF-β are not merely core components of their respective pathways but also serve as shared signaling hub nodes connecting the immune-fibrosis-pain axes. Future therapeutic strategies should move beyond single-target inhibition, shifting towards network pharmacology interventions targeting these key hub nodes, or using combination therapies to disrupt the neuro-immune feedback loop, thereby breaking the disease’s domino effect.

## 7 Other bioactive substances

### 7.1 Regulation of TRP channels by inflammatory

Transient receptor potential (TRP) channels that exhibit unique responses to temperature are referred to as thermo-TRP channels. Among all the thermal TRP channels, TRPV1, TRPV2, TRPV3, TRPV4, TRPM8, and TRPA1 are expressed in specific subpopulations of sensory neurons within the dorsal root ganglia (DRG). These TRP channels are indirectly triggered by inflammatory mediators such as PGE2, bradykinin, ATP, NGF, and cytokines released during injury. Activation of GPCRs or tyrosine kinase receptors sensitizes TRPV1, increasing excitability, while simultaneously desensitizing the innocuous cold sensor TRPM8 (135–137).

Tonyali et al. found increased NGF and GDNF expression in the bladder and urethral tissues of IC/PBS patients, which caused sensitization of bladder afferent nociceptors. Moreover, ARTN, a GDNF ligand, displayed mRNA levels in IC patients that were five times higher than NGF or GDNF (138). Additionally, artemin (ARTN), a member of the GDNF ligand family, exhibited mRNA levels in IC that were up to five times higher than those of NGF or GDNF (139, 140). ARTN enhances the opening time and intensity of TRPV1 and TRPA1 ion channels, thereby lowering the pain threshold. Research has shown that in a cyclophosphamide (CYP)-induced cystitis mouse model, there is an increase in bladder-derived ARTN and NGF as well as TRPA1 expression in bladder afferents. ARTN sensitizes Ca^2+^ signaling induced by mustard oil (MO) in bladder afferents, leading to bladder pain and persistent hypersensitivity (141). Furthermore, neutralizing antibodies against ARTN (α-ARTN) effectively blocked bladder hypersensitivity and reversed CYP-induced hypersensitivity *in vivo*, restoring normal expression levels of TRPA1 in bladder afferents and pERK in the spinal cord, resulting in significant alleviation of bladder pain (142).

Moreover, Yang et al. revealed that blocking adenosine A2a receptors reduces bladder overactivity and hypersensitivity caused by CYP-induced cystitis by suppressing TRPV1. The research observed an upregulation of A2a receptor expression within the bladder afferent pathway following CYP treatment. By targeting these receptors, bladder sensitivity decreased significantly, leading to a marked reduction in urinary frequency (p < 0. 01). The von Frey test also confirmed a notable decrease in pain sensitivity (p < 0. 05). Additionally, TRPV1 receptors showed increased expression in the bladder tissues of the CYP model, co-localized with A2a receptors in L6-S1 DRG neurons. When healthy animals were administered an A2a receptor agonist intrathecally, it induced bladder overactivity; however, pre-treatment with a TRPV1 antagonist significantly mitigated these symptoms (p < 0. 01), establishing a direct link between A2a and TRPV1 activation. The findings suggest that A2a receptor upregulation in bladder afferents plays a critical role in driving overactivity and hypersensitivity during CYP-induced cystitis through its interaction with TRPV1 (143). Chronic inflammation caused by cyclophosphamide raises extracellular adenosine levels, thereby overstimulating A2a receptors. This overstimulation heightens TRPV1 sensitivity in DRG neurons via the cAMP/PKA signaling pathway, leading to increased intracellular calcium levels and promoting glutamate release to the spinal cord. Consequently, this chain of events exacerbates bladder overactivity and hypersensitivity. Targeting A2a receptors in bladder afferents offers a promising strategy to alleviate these symptoms by modulating TRPV1 during CYP-induced cystitis.

### 7.2 APF

Anti-proliferative factor (APF) is a unique glycopeptide recognized as a urinary biomarker and potential mediator in interstitial cystitis/bladder pain syndrome (IC/BPS). APF exhibits several effects, including inhibiting cell growth, increasing cell permeability, and reducing the expression of proteins involved in forming intercellular junction complexes. In contrast, epidermal growth factor (EGF) acts as a potent mitogen for uroepithelial and smooth muscle cells, promoting proliferation and repair (144–149). Compared to the control group (urine from non-IC/PBS patients), the concentrations of APF and EGF in the urine of IC/PBS patients positively correlate with the presence of the disease. This is attributed to the presence of APF molecules, which inhibit the tissue repair process, leading to elevated EGF levels due to a prolonged state of damage.

In bladder epithelial cells, APF induces abnormal cellular signaling through the c-Jun transcription factor, which is involved in the JNK/SAPK pathway and contributes to the inhibition of cell growth (150). Concurrently, APF induces a significant decrease in c-Jun expression, resulting in a positive feedback loop that leads to growth inhibition and slow or impaired tissue repair in the bladder epithelium. However, overexpression of c-Jun can reverse the inhibitory effects of APF on cell growth (150, 151). Furthermore, APF promotes the phosphorylation of Akt and its target, glycogen synthase kinase (GSK) (152, 153), leading to downregulation of Akt and its downstream signaling pathways, thereby impairing tissue repair processes activated by the Akt pathway. This observation aligns with the slow tissue repair noted in IC/PBS cells. However, it remains unproven whether this downregulation of Akt in response to APF is dependent on the PI3K or PTEN pathways, with PTEN being a significant upstream inhibitor of the Akt signaling pathway (154, 155). Additionally, APF enhances the p53 signaling pathway, leading to inhibited cell proliferation (152). Activation of the p53 pathway is stimulated by stress signals such as DNA damage and oxidative stress, as well as by activated oncogenes (156–158), resulting in cell cycle arrest, cellular senescence, apoptosis, or repair of damaged DNA (159, 160). Treatment with APF in normal human uroepithelial cells and T24 human bladder cancer cells has been shown to increase p53 levels, indicating that APF enhances the expression of the p53 gene (152).

## 8 Therapeutic targets and clinical perspectives

### 8.1 Clinically relevant targets with translational potential

#### 8.1.1 Oxidative stress and ischemia-hypoxia

Targets: Reactive Oxygen Species (ROS) and Hypoxia-Inducible Factor-1α (HIF-1α).

Intervention Strategies: The use of antioxidants (e.g., N-acetylcysteine, NAC) to scavenge excess ROS has been shown to alleviate urinary frequency and pain symptoms in animal models. Although HIF-1α plays a protective role in the initial stages of ischemia-hypoxia, the pathological angiogenesis it mediates (resulting in abnormally fragile blood vessels) is a major contributor to mucosal bleeding in HIC patients. Therefore, hyperbaric oxygen therapy, which improves overall bladder oxygenation by addressing the root cause of ischemia-hypoxia, may be more desirable than completely blocking the HIF-1 pathway. Currently, NAC is readily available as a nutritional supplement, but its clinical efficacy in IC/BPS requires validation through large-scale trials. Hyperbaric oxygen therapy is already used as an experimental treatment for refractory IC/BPS in some medical centers.

#### 8.1.2 Immunity and inflammation

Targets: NLRP3 Inflammasome and Interleukin-1 (IL-1).

Intervention Strategies: NLRP3 is central to initiating the inflammatory cascade. Using inhibitors like dapansutrile in animal models significantly reduces inflammatory cell infiltration and bladder fibrosis. More direct clinical evidence comes from studies using IL-1 receptor antagonists (e.g., anakinra), which have shown significant improvements in urinary frequency, pelvic pain, and quality of life in IC patients. This provides strong preclinical and preliminary clinical evidence supporting therapies targeting IL-1β. Anakinra, already approved for rheumatoid arthritis, requires further multi-center clinical trials to confirm its efficacy in IC/BPS.

#### 8.1.3 Neuroinflammation and central sensitization

Targets: P2X7 Receptor, JAK Kinase, and NOTCH1 Receptor.

Intervention Strategies: These targets are critically involved in pain signal transduction and neuroinflammation. P2X7 antagonists effectively reduce bladder tissue damage and pain sensitivity in animal models. The application of JAK inhibitors (e.g., AG490) significantly decreases bladder hyperreflexia and hind paw sensitivity, confirming the crucial role of the JAK-STAT pathway in mediating IC-related pain. Furthermore, γ-secretase inhibitors (e.g., DAPT), which block NOTCH signaling, effectively reverse the decreased pain threshold in animal models. While these inhibitors are primarily in the preclinical research phase, they point towards promising directions for the development of novel analgesics.

### 8.2 Targets at the experimental stage

#### 8.2.1 Epithelial injury and repair

Targets: Vascular Endothelial Growth Factor (VEGF) and Antiproliferative Factor (APF).

Intervention Strategies: Anti-VEGF neutralizing antibodies (e.g., bevacizumab/B20) or anti-angiogenic tyrosine kinase inhibitors (e.g., axitinib), by blocking VEGF signaling, not only reduce pathological angiogenesis, edema, and hemorrhage but also decrease peripheral nerve density mediated by the interaction of VEGF with neuropilin (NRP), thereby alleviating pain. Conversely, interventions targeting APF aim to release its inhibitory effect on cell proliferation. Activating downstream effectors like c-Jun or modulating the p53 pathway could theoretically reverse the growth arrest induced by APF and promote epithelial repair. However, these strategies remain at the mechanistic exploration stage.

#### 8.2.2 Tissue fibrosis

Targets: Transforming Growth Factor-β (TGF-β) and Wnt/β-catenin pathways.

Intervention Strategies: These pathways are core drivers of organ fibrosis. While directly targeting TGF-β may have side effects due to its role in homeostasis, inhibiting its upstream activators (e.g., NLRP3) or downstream effectors (e.g., Smad) might be safer. For instance, curcumin inhibits the NLRP3 inflammasome, indirectly downregulating TGF-β/Smad signaling and reducing bladder fibrosis in animal models. Strategies targeting the Wnt pathway are more complex, given its dual role in tissue repair and fibrosis. Studies show that downregulation of certain *Wnt* genes in NHIC patients can paradoxically activate TGF-β signaling and promote fibrosis, suggesting that in specific subtypes, activating rather than inhibiting particular Wnt members (e.g., WNT11) might be beneficial.

In summary, targeted therapy for IC/BPS is evolving from a “one-size-fits-all” anti-inflammatory or analgesic approach towards “precision strikes” tailored to different disease subtypes (HIC vs. NHIC) and distinct pathological processes (epithelial injury, fibrosis, neuroinflammation). In the future, stratifying patients using biomarkers (e.g., urinary APF, cytokine profiles) and developing combination therapies that can simultaneously target multiple key pathways will be essential for achieving long-term, effective management of IC/BPS. .

## 9 Summary

Although interstitial cystitis/bladder pain syndrome (IC/BPS) is not life-threatening, its severe symptoms significantly diminish the quality of life for affected patients, causing considerable suffering. The etiology of IC/BPS remains unclear, and there is currently no straightforward diagnostic gold standard or long-term effective treatment regimen, which poses significant challenges for urologists. Signal transduction pathways are crucial for studying the pathophysiological processes of various diseases, and the detection and intervention of bioactive molecules within these pathways are essential tools for disease diagnosis and treatment. This article provides a retrospective analysis summarizing numerous IC-related signaling pathways and elucidates their pathophysiological roles, laying a theoretical foundation for researching the mechanisms of onset and developing long-lasting effective treatments (**Table 1**).

**Table 1:** Consolidated Signaling Pathways in IC/BPS.

Signaling pathway	Main function	Associated symptoms	Core molecules/biomarkers	Interventions/inhibitors (target)	Upstream pathway	Downstream pathway	Expression in IC	Ref.
HIF-1 Signaling Pathway	Regulates oxygen homeostasis, promotes capillary formation, reduces oxygen consumption, enhances oxygen transport, mitigates oxidative stress, and inhibits hypoxia-induced apoptosis and pyroptosis.	Bladder mucosal defects, capillary neogenesis	HIF-1α, HIF-1β, VEGF, G6PD, PGD	—	Chronic hypoxia	Pentose phosphate pathway	Upregulated in IC, particularly in Hunner’s ulcer areas	[20, 36, 43, 66, 68, 82, 84, 127, 142]
VEGF Signaling Pathway	Promotes angiogenesis, neurovascular growth, and inflammation, influencing central sensitization and bladder pain through interaction with NRP and integrins.	Urinary frequency, pelvic sensitivity, bladder pain	VEGF, VEGFR, NRP, TRPV1, PGP9.5	B20, anti-NRP (NRP)	HIF-1 Pathway	Neurovascular growth	Upregulated in urothelium, lamina propria, and intramural ganglia	[25, 32, 33, 37, 53, 58, 66, 70, 78, 85, 89, 93, 107, 129–132, 141, 159]
PI3K-mTOR Signaling Pathway	Regulates cell proliferation, differentiation, apoptosis, and nociception; alleviates pain by modulating CGRP and substance P; reduces bladder hypersensitivity and tissue fibrosis.	Bladder hypersensitivity, pelvic pain	PI3K, mTOR, CGRP, Substance P	LY294002 (PI3K), rapamycin (mTOR)	Growth factor signaling	Cell proliferation and repair	Upregulated in spinal neurons and dorsal horn during IC	[118, 120, 124, 135, 29, 91, 95, 138, 160]
NLR Signaling Pathway	Activates inflammasomes, promotes IL-1β production, induces pyroptosis, mediates inflammatory cell infiltration, and triggers fibrosis via TGF-β/Smad pathway.	Bladder pain, urinary frequency, bladder fibrosis	NLRP3, ASC, Caspase-1, IL-1β, CXCL10, TGF-β, Smad	DAP (NLRP3)*	ROS, pathogen products	TGF-β/Smad, EMT	Upregulated NLRP3 and related inflammatory markers in IC	[16, 26, 28, 41, 54, 86, 98, 101, 104, 106, 108, 114]
TGF-β Signaling Pathway	Promotes epithelial cell proliferation, tissue repair, and fibrosis; mediates EMT; regulates inflammation through iNOS and NO modulation.	Tissue fibrosis, pain, inflammation	TGF-β1, iNOS, NO, Smad, MMPs	—	NLRP3 Activation	EMT, MAPK signaling	Elevated TGF-β1 and fibrosis markers in bladder tissues	[17, 34, 45, 59, 62, 63, 74, 76, 90, 99, 100, 112, 123, 128, 136, 139, 140, 148, 150]
Wnt Signaling Pathway	Maintains urothelial homeostasis, promotes epithelial proliferation and tissue repair, and mediates fibrosis through EMT and extracellular matrix remodeling.	Bladder fibrosis, epithelial shedding	Wnt, β-catenin, SLUG, ZEB1, TWIST	—	Damage-induced activation	EMT, extracellular matrix remodeling	Altered Wnt expression, reduced in NHIC; associated with fibrosis	[18, 19, 39, 47, 57, 69, 81, 113, 149]
Chemokine Signaling Pathway	Mediates inflammatory cell migration, neovascularization, and immune activation; regulates inflammation, apoptosis, and tissue damage via MAPK, PI3K, and NF-κB pathways.	Inflammation, bladder pain	CCL2, CCL5, CXCL10, CXCR3, CXCR4, CCR5	TAK-779 (CCR5)	Mechanical injury, infection	MAPK, PI3K, NF-κB	Upregulated chemokines and receptors in blood, urine, and bladder tissues	[15, 30, 31, 60, 119, 121, 125, 137]
JAK-STAT Signaling Pathway	Mediates cytokine and growth factor signaling; promotes inflammation and fibrosis; alleviates IC symptoms when inhibited.	Bladder hyperreflexia, pain, fibrosis	JAK, STAT, MicroRNA-132, MicroRNA-495	AG490 (JAK)	Cytokine signaling	Transcription of pro-inflammatory genes	Elevated JAK3 and STAT activation in bladder tissues	[42, 67, 79, 94, 109]
Toll-Like Receptor (TLR) Pathway	Activates immune responses via MyD88-dependent and -independent pathways; mediates inflammation and neuropathic pain through cytokine release and NF-κB activation.	Pelvic pain, central sensitization	TLR2, TLR4, NF-κB, IL-1β, IL-6, TNF-α	—	Pathogen-derived ligands	NF-κB, MAPK signaling	Upregulated TLR2, TLR4 in bladder tissues and blood	[23, 35, 55, 80, 87, 103, 110, 111, 116, 153]
Neurotrophic Factor-TNF-α/NF-κB Pathway	Regulates neuronal differentiation and survival; promotes neuroinflammation and persistent pain through TNF-α and IL-1β signaling.	Neuropathic pain, inflammation, central sensitization	NGF, BDNF, TNF-α, NF-κB, IL-1β	PDTC (NF-κB)	TNFR1 Activation	MAPK, PI3K, PLC signaling	Upregulated TNF-α, IL-1β, and NF-κB signaling in SDH and hippocampus of IC models	[27, 40, 51, 56, 72, 75, 77, 83, 102]
NOTCH Signaling Pathway	Regulates cell proliferation and differentiation; promotes microglial activation and neuroinflammation, mediating mechanical allodynia and pain in IC.	Pelvic pain, neuroinflammation	NOTCH1, NICD, Iba1, p-p38, TNF-α, IL-1β	DAPT (NOTCH1)	Gamma-secretase cleavage	Microglial activation	Upregulated NOTCH1 and NICD in spinal dorsal horn of IC models	[122]

*The content in parentheses indicates inhibitors corresponding to the preceding molecules.

Among the signaling pathways discussed, those with the strongest human-based evidence include the HIF-1, VEGF, NLRP3, and TGF-β pathways, each supported by clinical biopsy data, urinary biomarker studies, or interventional responses in IC/BPS patients. Promising biomarker candidates include urinary APF (anti-proliferative factor), chemokines (e.g., CXCL10, CCL2), nerve growth factor (NGF), and components of the NLRP3 inflammasome, which correlate with symptom severity and disease subtype. However, critical knowledge gaps remain. Future research should prioritize patient stratification by phenotype (e.g., HIC vs. NHIC), as these subtypes exhibit distinct molecular signatures and treatment responses. Longitudinal studies are urgently needed to determine whether pathway alterations are causal or secondary to chronic inflammation. Additionally, sex-specific differences in pathway activation—particularly in TGF-β and NO signaling—warrant deeper investigation to explain the female predominance of IC/BPS. Bridging these gaps will be essential for the development of targeted diagnostics and individualized therapeutic strategies.
