# Supplementary material for: Signaling pathways related to interstitial cystitis
Source: Front Immunol. 2026 Apr 23;17:1774072. doi: 10.3389/fimmu.2026.1774072 (PMC13149192; doi:10.3389/fimmu.2026.1774072)
Supplement: Supplementary file 1 [file Table1.docx]

**Signaling Pathways Related to Interstitial Cystitis**

**Authors.** Wang Haowen^1^†, Liu Shuang^1^†, Sun Yuan^2^ ,Wang Lina^1^

**Corresponding author:**

Professor: Wang Lina

Address: Zhongshan Road No. 222, Dalian, China.

Tel. : +86-411-83635963-2104.

E-mail address: [doctor_dlw@163. com](mailto:doctor_dlw@163.com)

Professor: Sun Yuan

Address: Liaoning Laboratory of Cancer Genomics and Department of Cell Biology, Dalian Medical University, Dalian, China.

Tel. : +86-411-86110318

E-mail address: sunyuandl@dmu. edu. Cn

† These authors have contributed equally to this work

**Affiliations:**

1. Department of Urology, First Affiliated Hospital of Dalian Medical University, Zhongshan Road No. 222, Dalian, 116021, China. wanghaowen0820@163. com and liushuang@163. com.
2. Liaoning Laboratory of Cancer Genomics and Department of Cell Biology, Dalian Medical University, Dalian, 116021,China.
