# Supplementary material for: Signaling pathways related to interstitial cystitis
Source: Front Immunol. 2026 Apr 23;17:1774072. doi: 10.3389/fimmu.2026.1774072 (PMC13149192; doi:10.3389/fimmu.2026.1774072)
Supplement: Supplementary file 2 [file Table2.docx]

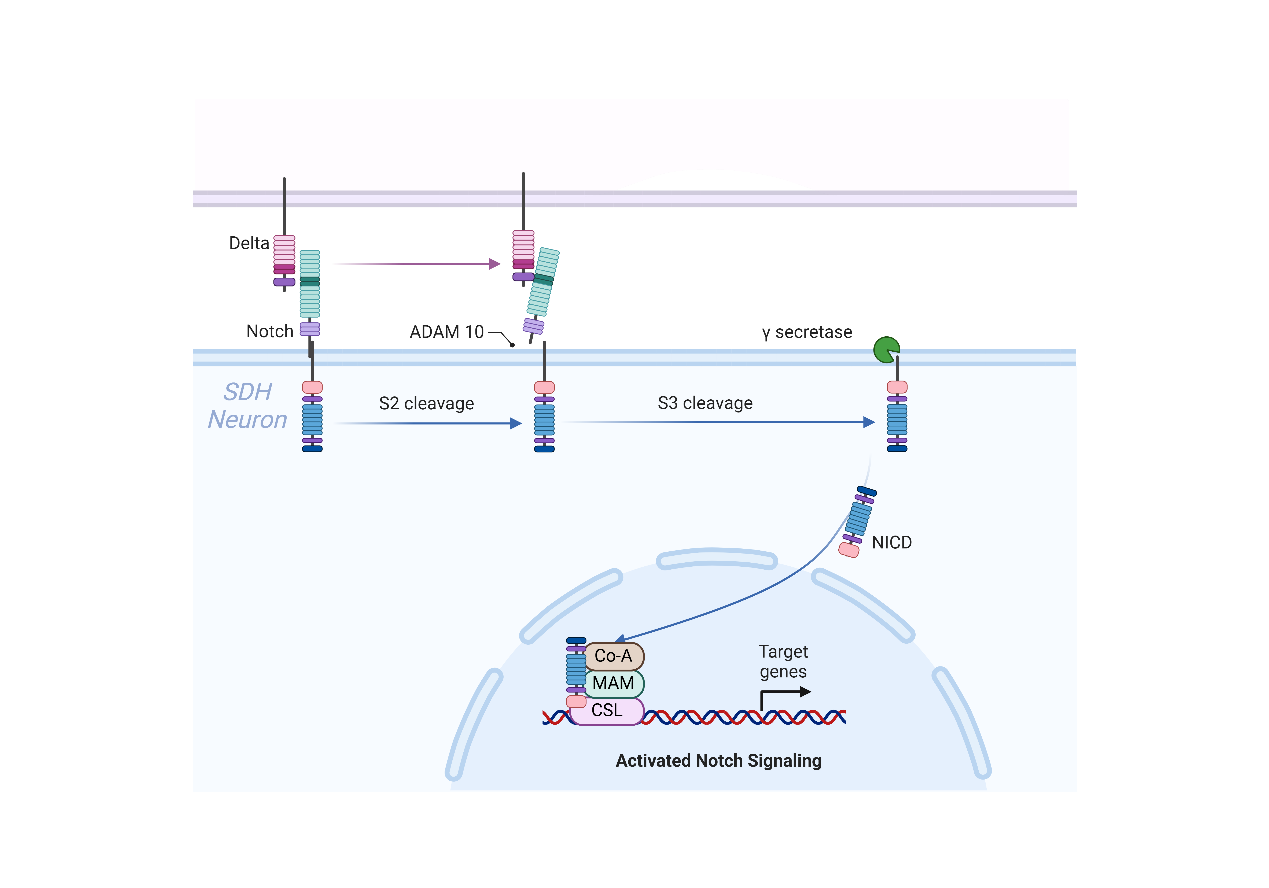


The diagram illustrates the **Notch signaling pathway** and its role in neuroinflammation and mechanical allodynia associated with **interstitial cystitis/bladder pain syndrome (IC/BPS)**. Notch signaling is initiated when the Delta ligand binds to the Notch receptor, triggering S2 cleavage by ADAM10 and S3 cleavage by γ-secretase. This releases the Notch intracellular domain (NICD), which translocates to the nucleus to activate target genes. In IC, Notch1 upregulation in the spinal dorsal horn (SDH) promotes microglial activation and inflammatory cytokine production (e.g., TNF-α, IL-1β), reducing pain thresholds and contributing to pelvic pain. Inhibition of Notch1 with γ-secretase inhibitors alleviates neuroinflammation and pain.
