# Supplementary material for: Signaling pathways related to interstitial cystitis
Source: Front Immunol. 2026 Apr 23;17:1774072. doi: 10.3389/fimmu.2026.1774072 (PMC13149192; doi:10.3389/fimmu.2026.1774072)
Supplement: Supplementary file 3 [file Table3.docx]

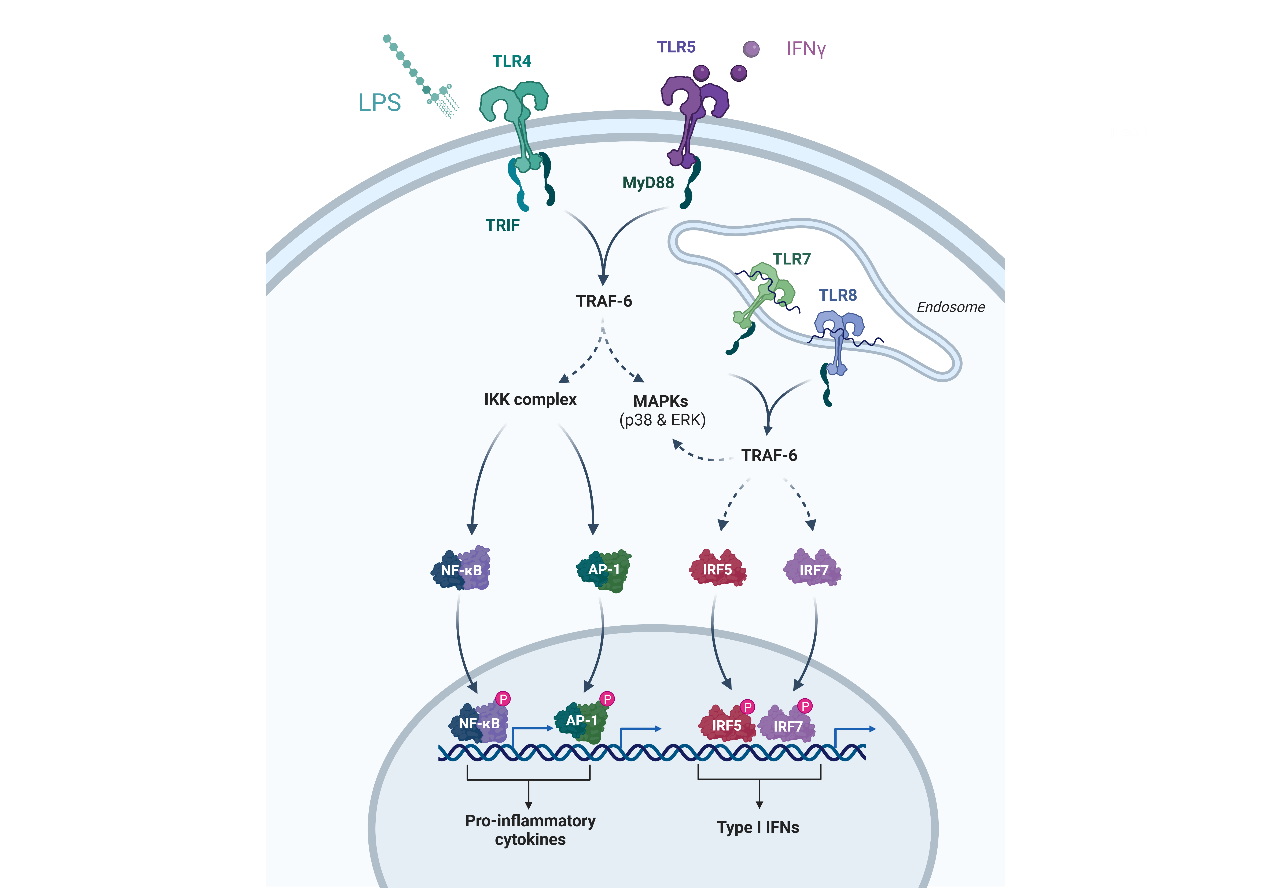


The diagram illustrates the **Toll-Like Receptor (TLR) signaling pathway** and its role in inflammation and pain in **interstitial cystitis (IC)**. TLR4, activated by LPS, triggers MyD88- and TRIF-dependent pathways, leading to the activation of NF-κB, AP-1, and MAPKs (p38, ERK). These pathways result in the production of pro-inflammatory cytokines (IL-1β, IL-6, TNF-α). Endosomal TLR7/8, activated by IFNγ, signals through MyD88, inducing IRF5/7-mediated type I IFN production. In IC, upregulation of TLR4 and TLR7 correlates with inflammatory cytokine release, bladder pain, and central sensitization. TLR2 and TLR4 hyperactivity have been implicated in neuropathic pain pathways, linking TLR signaling to chronic pelvic pain and immune-mediated inflammation.
