# Supplementary material for: Signaling pathways related to interstitial cystitis
Source: Front Immunol. 2026 Apr 23;17:1774072. doi: 10.3389/fimmu.2026.1774072 (PMC13149192; doi:10.3389/fimmu.2026.1774072)
Supplement: Supplementary file 4 [file Table4.docx]

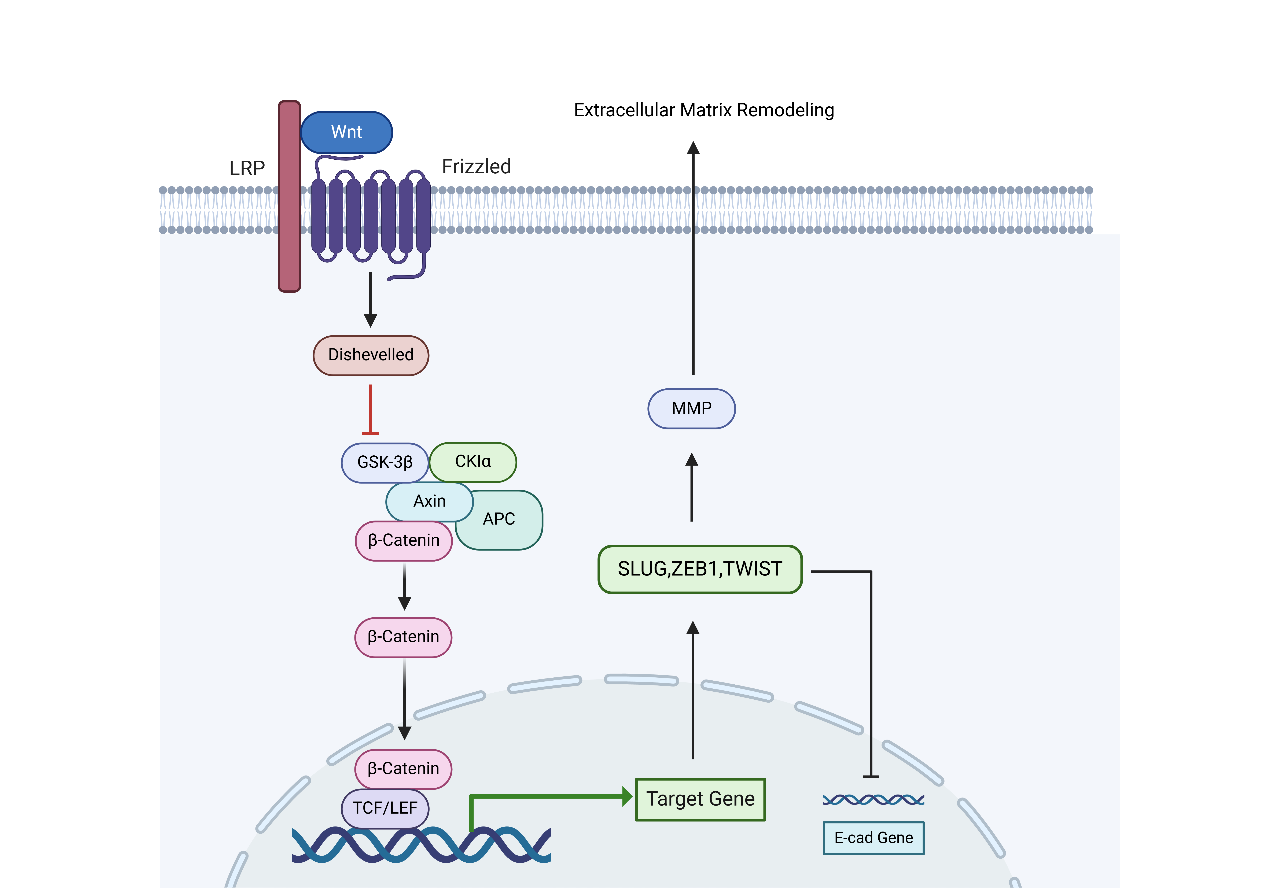


The diagram illustrates the **Wnt/β-catenin signaling pathway** and its involvement in tissue repair and epithelial-mesenchymal transition (EMT) in **interstitial cystitis (IC)**. Wnt binds to Frizzled and LRP receptors, activating Dishevelled, which inhibits GSK-3β, stabilizing β-catenin. β-catenin translocates to the nucleus, activating target genes via TCF/LEF, leading to epithelial cell proliferation and tissue repair. Additionally, Wnt signaling induces EMT by upregulating mesenchymal markers (SLUG, ZEB1, TWIST) and promoting matrix metalloproteinase (MMP) expression, suppressing epithelial markers like E-cadherin. In IC, Wnt pathway dysfunction or gene silencing can trigger fibrosis through TGF-β activation, exacerbating bladder tissue damage and chronic inflammation.
