# Supplementary material for: Signaling pathways related to interstitial cystitis
Source: Front Immunol. 2026 Apr 23;17:1774072. doi: 10.3389/fimmu.2026.1774072 (PMC13149192; doi:10.3389/fimmu.2026.1774072)
Supplement: Supplementary file 5 [file Table5.docx]

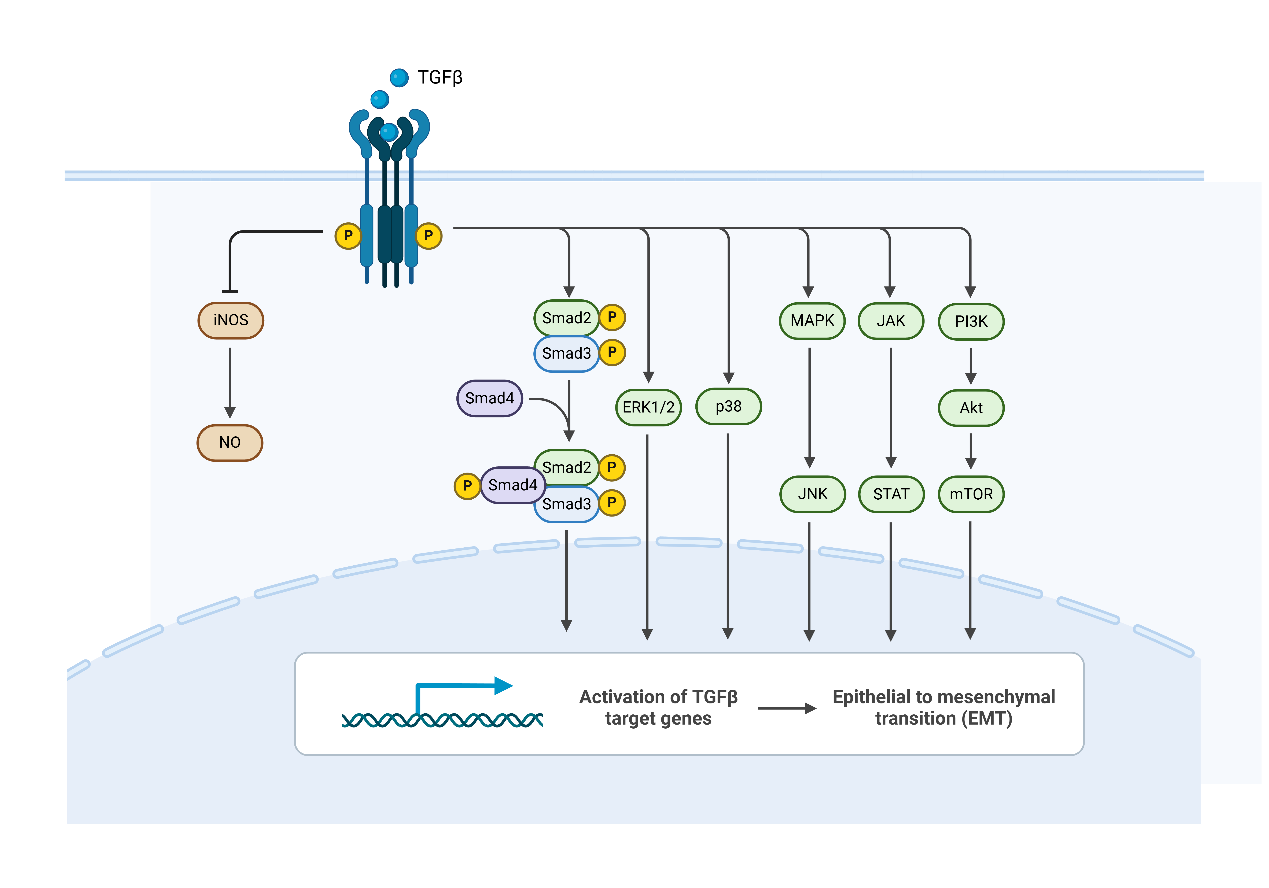


The diagram illustrates the **TGF-β signaling pathway** and its role in epithelial-mesenchymal transition (EMT) and inflammation in **interstitial cystitis (IC)**. TGF-β binds to its receptors, phosphorylating Smad2/3, which forms a complex with Smad4 to activate downstream target genes, leading to EMT. Non-canonical pathways, such as MAPK (ERK1/2, p38, JNK), JAK/STAT, and PI3K/Akt/mTOR, are also activated, promoting cell proliferation, inflammation, and tissue repair. In IC, upregulation of TGF-β contributes to bladder epithelial damage, mast cell activation, and fibrosis. TGF-β also suppresses iNOS and NO production, reducing inflammation, while its activation promotes tissue remodeling and chronic bladder dysfunction through EMT processes.
