# Supplementary material for: Signaling pathways related to interstitial cystitis
Source: Front Immunol. 2026 Apr 23;17:1774072. doi: 10.3389/fimmu.2026.1774072 (PMC13149192; doi:10.3389/fimmu.2026.1774072)
Supplement: Supplementary file 6 [file Table6.docx]

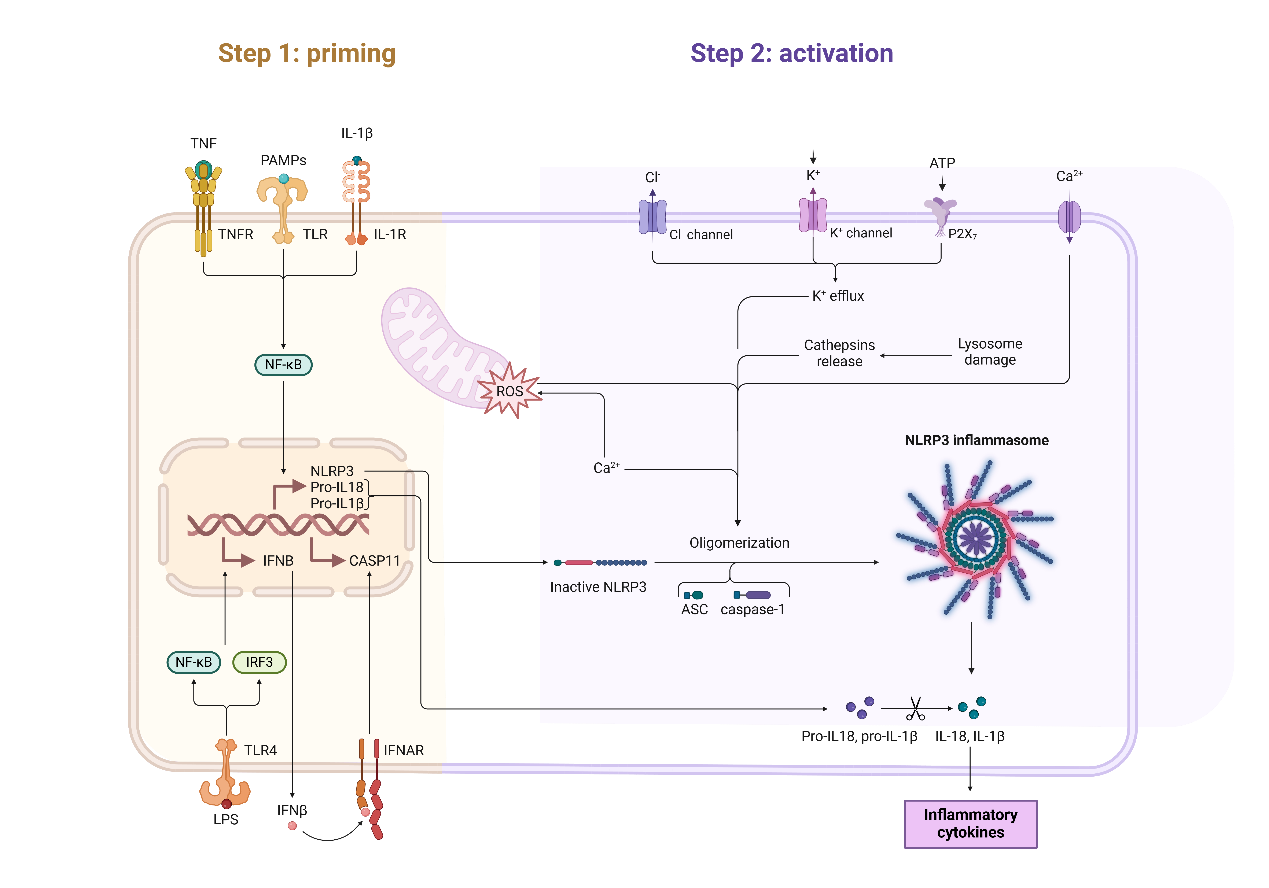


The diagram illustrates the **NLRP3 inflammasome activation pathway** and its role in inflammation and fibrosis in **interstitial cystitis (IC)**. The process occurs in two steps: **priming** and **activation**. In step 1, priming begins with the activation of NF-κB via TNF, TLRs, or IL-1 receptors, leading to the transcription of NLRP3, pro-IL-1β, and pro-IL-18. In step 2, cellular stress signals, including ROS production, K⁺ efflux, lysosome damage, and Ca²⁺ influx, activate the NLRP3 inflammasome. Oligomerized NLRP3 activates caspase-1, cleaving pro-IL-1β and pro-IL-18 into active inflammatory cytokines, which induce pyroptosis and trigger inflammatory responses. In IC, this pathway promotes inflammatory cell infiltration, cytokine release, bladder pain, and tissue fibrosis.
