# Supplementary material for: Signaling pathways related to interstitial cystitis
Source: Front Immunol. 2026 Apr 23;17:1774072. doi: 10.3389/fimmu.2026.1774072 (PMC13149192; doi:10.3389/fimmu.2026.1774072)
Supplement: Supplementary file 7 [file Table7.docx]

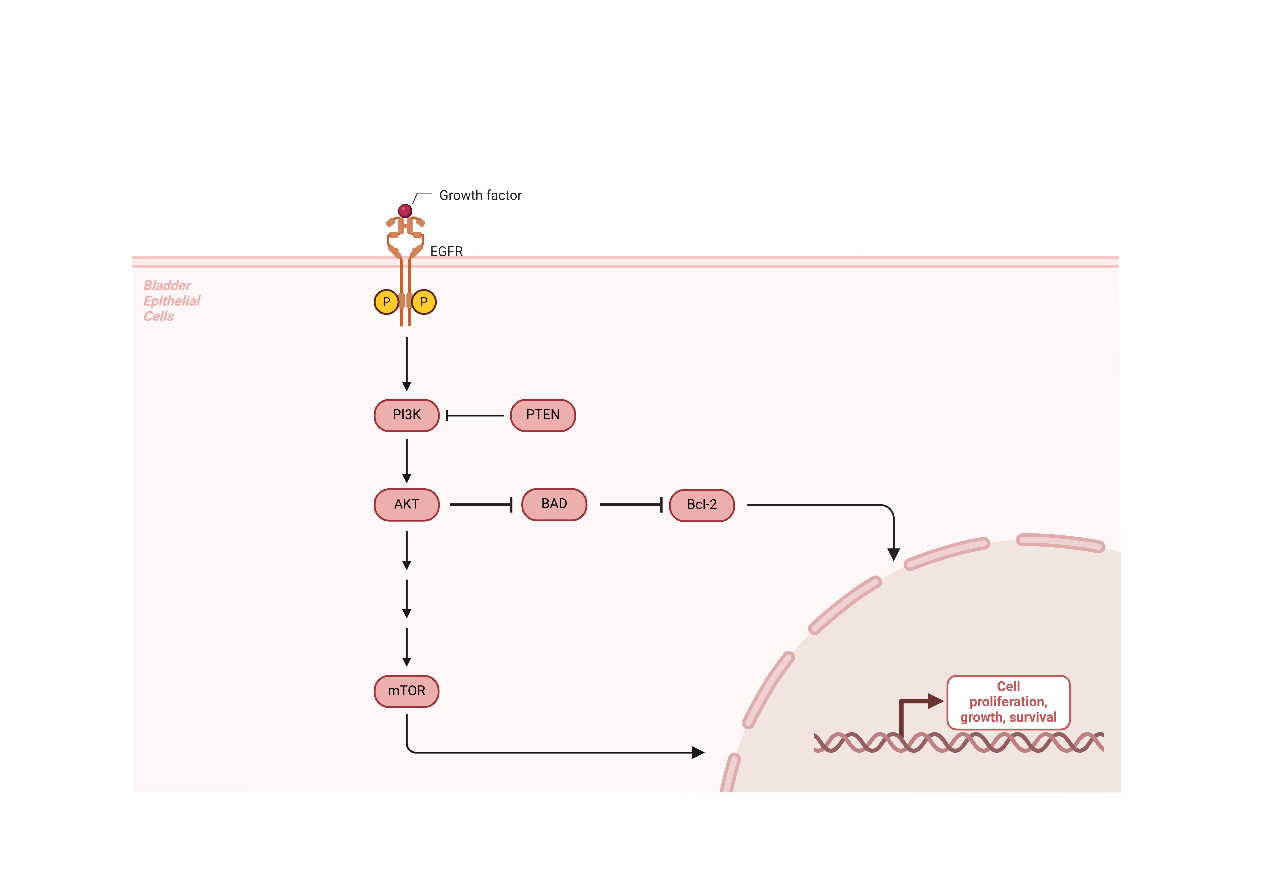


The diagram illustrates the **PI3K-Akt/mTOR signaling pathway** and its role in promoting cell proliferation, survival, and repair in **interstitial cystitis (IC)**. Growth factors such as EGF bind to their receptors (EGFR), activating PI3K, which phosphorylates Akt. Activated Akt inhibits pro-apoptotic proteins like BAD and promotes anti-apoptotic proteins like Bcl-2, enhancing cell survival. Akt also activates mTOR, which stimulates cell proliferation, growth, and tissue repair through downstream transcription factors like HIF-1α. In IC, this pathway plays a key role in repairing damaged bladder urothelium, reducing apoptosis, and mitigating inflammation, thus contributing to bladder epithelial regeneration and fibrosis prevention.
