# Supplementary material for: Signaling pathways related to interstitial cystitis
Source: Front Immunol. 2026 Apr 23;17:1774072. doi: 10.3389/fimmu.2026.1774072 (PMC13149192; doi:10.3389/fimmu.2026.1774072)
Supplement: Supplementary file 8 [file Table8.docx]

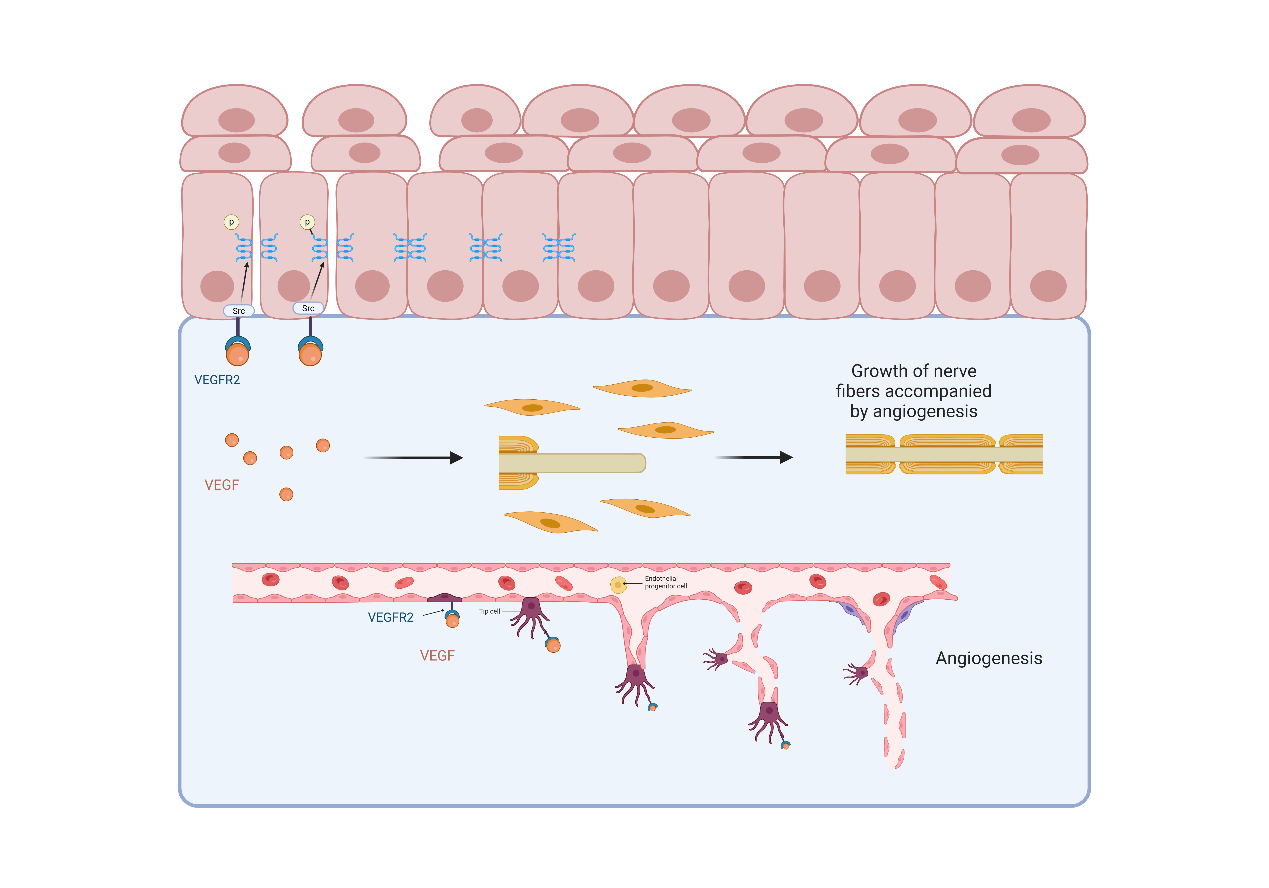


The diagram highlights the role of the **VEGF signaling pathway** in angiogenesis and epithelial injury, particularly in the context of **interstitial cystitis (IC)**. VEGF binds to VEGFR2 on the bladder urothelial cells, activating downstream pathways such as Src kinase, which phosphorylates junctional proteins like ZO-1. This disrupts tight junction integrity, increasing urothelial permeability and allowing toxic substances to penetrate the subepithelial layers, contributing to epithelial damage and pain. VEGF-induced angiogenesis promotes new capillary formation but results in fragile vasculature, exacerbating petechial bleeding observed in IC. Additionally, VEGF stimulates nerve fiber growth, linking angiogenesis to bladder pain hypersensitivity.
