# Supplementary material for: Signaling pathways related to interstitial cystitis
Source: Front Immunol. 2026 Apr 23;17:1774072. doi: 10.3389/fimmu.2026.1774072 (PMC13149192; doi:10.3389/fimmu.2026.1774072)
Supplement: Supplementary file 9 [file Table9.docx]

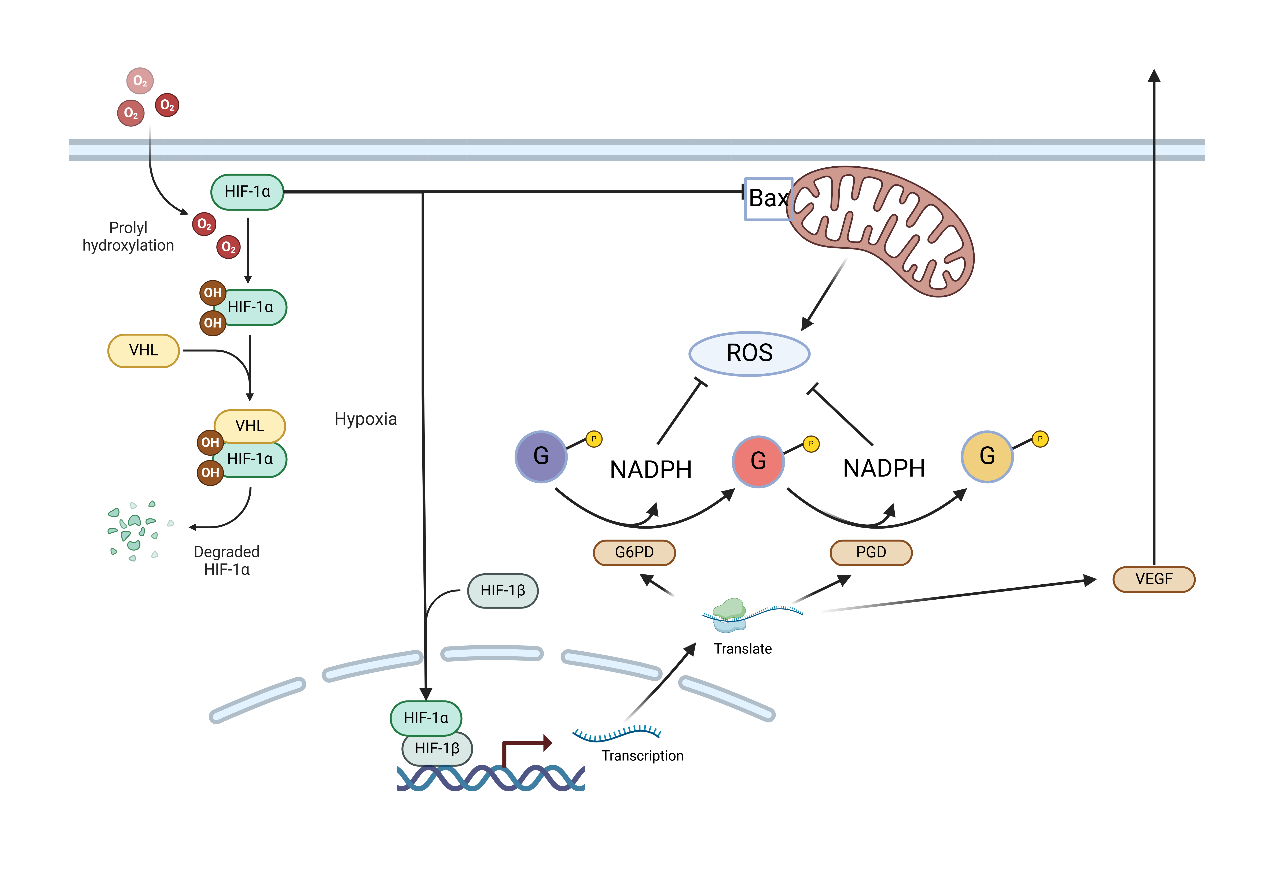


The diagram depicts the HIF-1 signaling pathway and its critical role in oxygen regulation and tissue protection, particularly in conditions like interstitial cystitis (IC). Under normoxia, HIF-1α undergoes hydroxylation, ubiquitination via VHL, and subsequent degradation. In hypoxic conditions, HIF-1α stabilizes, translocates to the nucleus, and pairs with HIF-1β to activate target genes such as VEGF, promoting angiogenesis and cellular survival. In IC, chronic bladder ischemia and hypoxia result in elevated HIF-1α expression, especially in ulcerated regions, contributing to capillary regeneration, reducing oxidative stress, and preventing hypoxia-induced apoptosis and epithelial injury. This pathway is crucial for mitigating bladder tissue damage seen in IC.
