# Supplementary material for: Signaling pathways related to interstitial cystitis
Source: Front Immunol. 2026 Apr 23;17:1774072. doi: 10.3389/fimmu.2026.1774072 (PMC13149192; doi:10.3389/fimmu.2026.1774072)
Supplement: Supplementary file 10 [file Table10.docx]

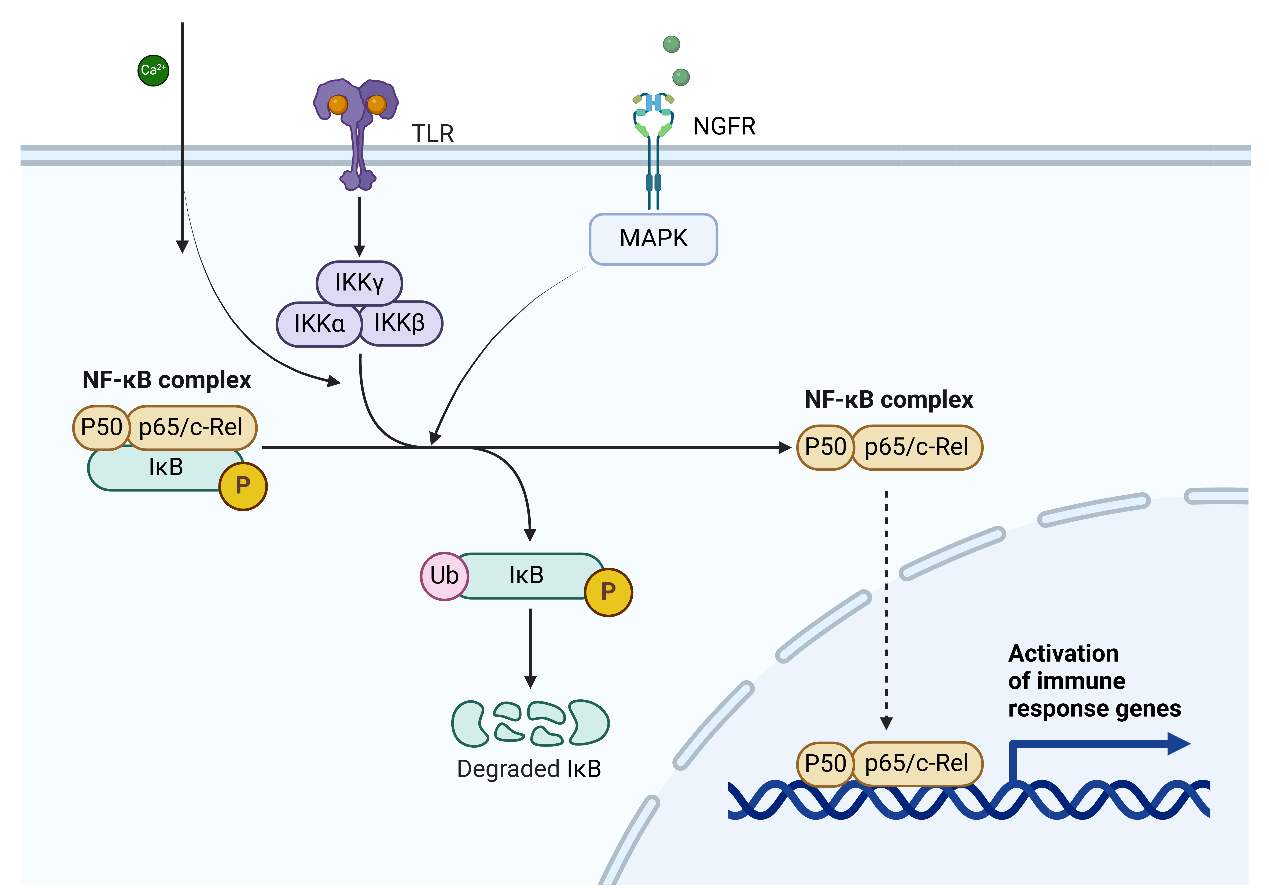


This figure illustrates the NF-κB signaling pathway, a critical pro-inflammatory mechanism with significant involvement in interstitial cystitis (IC). Toll-like receptor (TLR) activation leads to the recruitment of the IKK complex (IKKα, IKKβ, and IKKγ), which phosphorylates the inhibitory protein IκB. Phosphorylated IκB undergoes ubiquitination and proteasomal degradation, releasing the NF-κB complex (p50/p65). The liberated NF-κB translocates to the nucleus, where it activates the transcription of pro-inflammatory genes, including TNF-α and IL-1β. In interstitial cystitis, NF-κB activation in the spinal dorsal horn (SDH) and surrounding tissues upregulates TNF-α, IL-1β, and MAPK signaling markers such as p-p38 and p-JNK, leading to neuroinflammation. This neuroinflammation exacerbates pain hypersensitivity, lowers pain thresholds, and contributes to the chronic pain associated with IC. Furthermore, the persistent activation of NF-κB creates a self-amplifying inflammatory loop, perpetuating the release of cytokines and worsening symptoms in IC.
